# Beyond livestock carrying capacity in the Sahelian and Sudanian zones of West Africa

**DOI:** 10.1038/s41598-021-01706-4

**Published:** 2021-11-11

**Authors:** Jaber Rahimi, Edwin Haas, Rüdiger Grote, David Kraus, Andrew Smerald, Patrick Laux, John Goopy, Klaus Butterbach-Bahl

**Affiliations:** 1 Institute of Meteorology and Climate Research, Atmospheric Environmental Research (IMK-IFU), Karlsruhe Institute of Technology (KIT), Garmisch-Partenkirchen, Germany; 2 Mazingira Centre, International Livestock Research Institute (ILRI), Nairobi, Kenya

**Keywords:** Climate change, Climate-change impacts

## Abstract

We applied the process-based model, LandscapeDNDC, to estimate feed availability in the Sahelian and Sudanian agro-ecological zones of West Africa as a basis for calculating the regional Livestock Carrying Capacity (LCC). Comparison of the energy supply (S) from feed resources, including natural pasture, browse, and crop residues, with energy demand (D) of the livestock population for the period 1981–2020 allowed us to assess regional surpluses (S > D) or deficits (S < D) in feed availability. We show that in the last 40 years a large-scale shift from surplus to deficit has occurred. While during 1981–1990 only 27% of the area exceeded the LCC, it was 72% for the period 2011–2020. This was caused by a reduction in the total feed supply of ~ 8% and an increase in feed demand of ~ 37% per-decade, driven by climate change and increased livestock population, respectively. Overall, the S/D decreased from ~ 2.6 (surplus) in 1981 to ~ 0.5 (deficit) in 2019, with a north–south gradient of increasing S/D. As climate change continues and feed availability may likely further shrink, pastoralists either need to source external feed or significantly reduce livestock numbers to avoid overgrazing, land degradation, and any further conflicts for resources.

## Introduction

The ruminant livestock population in West Africa has expanded rapidly in recent decades (6–7 times greater than in 1961^1^) and forms an important part of the smallholder-dominated economy^2^. Most livestock are reared in pastoral and mixed crop-livestock systems, where feed is predominantly supplied from the locality or through transhumance movement^3^. In this study we examined whether the regional feed supply is sufficient to sustainably support the livestock population, disregarding transhumance. This will indicate where negative consequences of overgrazing have occurred or are likely to occur in the future^4^ and who is likely to bear the economic costs of extra feed imports (while in many cases smallholders do not have the economic strength to buy feed or even do not have market access). Identifying imbalances between regional feed supply and demand is crucial to decide, where ecological and economic mitigation strategies should be directed. It will also help in identifying potential for local trade in feed, which in turn may enhance the efficiency and profitability of livestock production in the area^5^. Therefore, our study is focused on determining the spatially- and temporally-resolved Livestock Carrying Capacity (LCC), defined here as the maximum stocking rate the ecosystem can support on a self-sufficient sustainable basis (i.e., long-term without resulting in environmental degradation of soils and vegetation cover)^6–9^. In particular, we were interested in assessing temporal and spatial changes of LCC and if these changes may be related to environmental and demographical changes (e.g., increase in livestock numbers, expansion of arable land, deforestation, desertification).

Changes in the LCC over time depend on a number of long-term drivers, either from supply-side (natural and managed ecosystems' productivity) or demand-side (demand on livestock products and other services provided by livestock). On the supply-side, hydro-climatic conditions are important determinants of productivity of ecosystems (natural and managed). Of particular importance in mostly semi-arid regions such as West Africa, is the seasonal distribution of precipitation^10–12^. Since the end of the 1970s, the annual temperature over West Africa has significantly increased (*P* < 0.01) by 0.2–0.8 °C^13,14^ and also rain patterns have changed, with the Sahelian zone becoming wetter and the southern parts of West Africa becoming drier. Furthermore, the precipitation frequency has changed and the intensity of extreme events has increased^14–17^, resulting in more droughts, floods, and heat waves^18,19^. All these changes can be expected to lead to altered biomass production.

Land-use and land-management (e.g. crop selection, soil-fertility management, grazing intensity) changes are additional important drivers of shifts in ecosystem’s functions and services. In recent decades, conversion of savannas, woodlands, and forests to agricultural land has been a prominent feature of land-use change^20–22^, but it is unclear whether this has resulted in increased feed availability for livestock.

On the demand side, high population growth has put pressure on agricultural production systems^23^, resulting in a remarkable increase in demands for livestock products, which was likely the main driver of the documented increase in livestock population. Furthermore, other reasons for livestock population increases could be due to greater demand for livestock’s function as savings repositories, producers of manure or providers of traction. According to FAOSTAT (Food and Agriculture Organization of United Nations Statistics) data from 2018^1^, West African livestock populations, (circa 80 million cattle, 123 million sheep and 178 million goats), are exceptionally large by Sub-Saharan Africa (SSA) standards^24^. In 2019, livestock density approached ~ 16 cattle, ~ 24 sheep, and ~ 35 goats per km^2^, respectively, while the average livestock density in other regions of Africa is ~ 11 of each cattle, sheep, and goat per km^2^.

Various approaches have been used to studying LCC in different regions of the world (e.g. for Mozambique^25^; Uganda^26^; Australia^27^; China^28^; South Africa^29^; Sudan^30^; Ethiopia^31^). In West Africa, these approaches include the use of remote sensing to compare feed supply and livestock demand^32,33^, as the conduct of surveys and statistical analyses^34,35^. Complementary to methods that refer more to the description of the current state of the system, process-based models allowing to simulate biomass production may be used to explore the underlying mechanisms of the system and to assess past and future climate and land use change effects on feed availability. One study^36^, uses a global rangeland model, called G-Range, to calculate the global herbaceous biomass production, but does not explicitly calculate the LCC. It predicts that under Representative Concentration Pathway (RCP) 8.5 emission scenario of IPCC Fifth Assessment Report (AR5), corresponds to a high greenhouse gas emissions pathway, the herbaceous biomass production in SSA in 2050 will be 23% lower than in 2000, and that this will threaten the future livestock population. This points to the importance of performing a more detailed study that includes all the commonly used feeds (i.e. crop residues and browse plus grass and forbs) and to consider the livestock demand and its fluctuations, so as to determine the LCC at a fine-grained scale.

In this study, we use the process-based biogeochemical model LandscapeDNDC to determine the feed production in the study area for the period 1981–2020 on a spatial grid of 0.1° (approx. 11 × 11 km). We provided a calculational framework with which to convert the main types of feed biomass into energetic values, and another to determine the energy required to support a given population of livestock. Using both frameworks, the total energy supply in a given area, S, can be compared to the total energy demand, D. Areas with S > D have a surplus of feed compared to their livestock population (ignoring wildlife), and are therefore below their LCC, while areas with S < D have a feed deficit, and are above their LCC, and the ratio S/D quantifies the magnitude of the surplus or deficit. This research will build detailed picture of where and to what extent the LCC has been exceeded and how this has changed over time, thus allowing the identification of ‘hotspots’ with regard to livestock populations outstripping their self-sufficient sustainable feed supply.

## Results and discussion

### Simulating biomass production at regional scale

**Figure 1:**
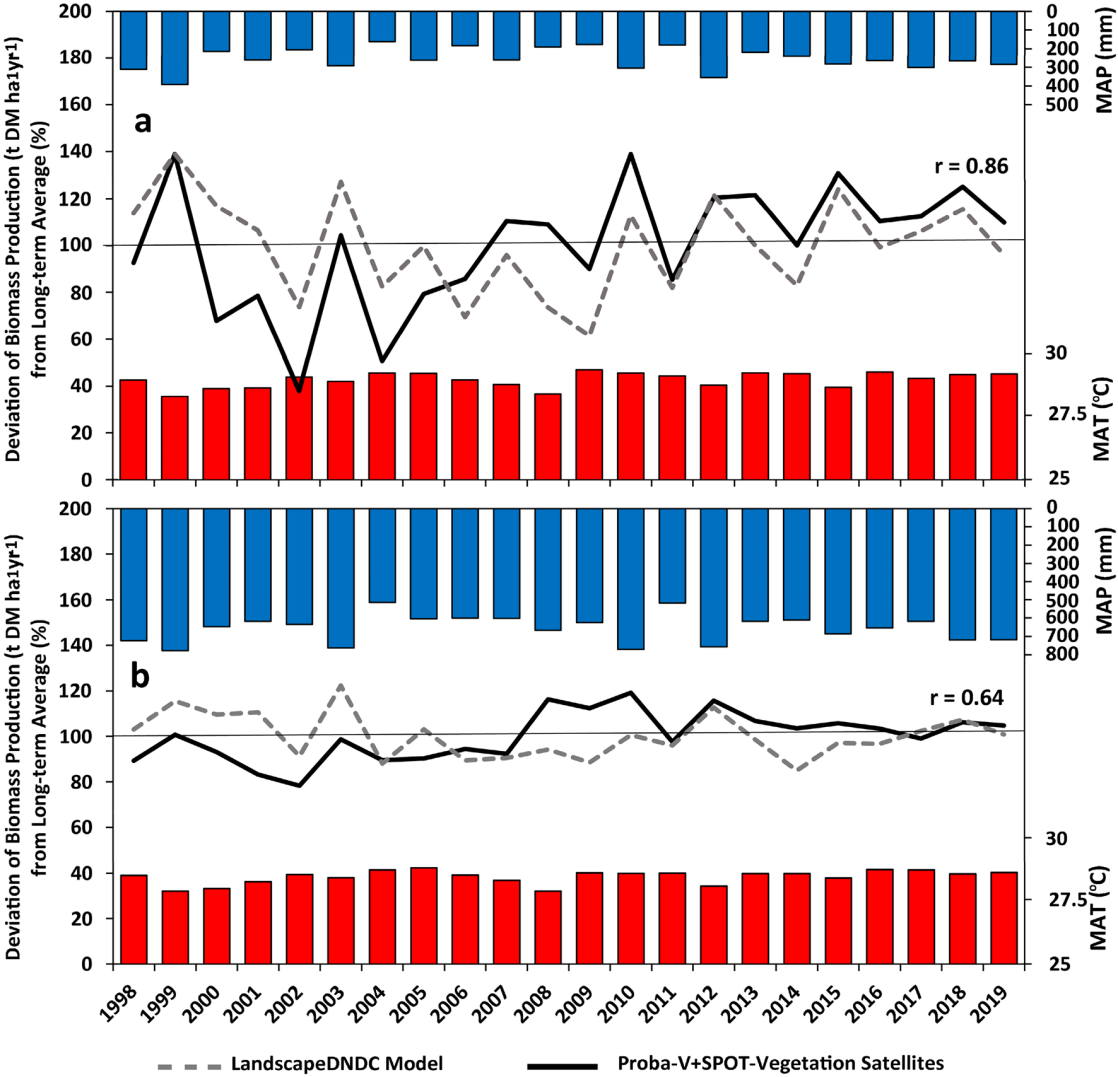
Mean Annual Precipitation (MAP), Mean Annual Temperature (MAT), as well as the deviation of total biomass production (t DM ha^−1^ yr^−1^) from long-term average (%), simulated with LandscapeDNDC model and estimated with the remote sensing data (ACF product, the Proba-V and the SPOT-vegetation satellites) over the Sahelian (**a**) and Sudanian (**b**) part of the study domain (the anomaly is shown on a scale between 0 and 200%). The figure was generated using Microsoft Excel 2016 (https://www.microsoft.com/de-de/microsoft-365/excel).

Biomass production was simulated using the process oriented model LandscapeDNDC, which has recently been assessed for its capability to simulate biomass dynamics of savannah type ecosystems and arable production systems in West Africa using available regional biomass production datasets and remote sensing data (see “ACF biomass production dataset” section)^37^. Here we compared total biomass production and its anomaly (comparison of total Dry Matter (DM) production of the current year with the average total DM production over the entire period) simulated with LandscapeDNDC and estimates based on remote sensing data of the ACF program (Action Contre La Faim or Action Against Hunger (AAH) program, see “ACF biomass production dataset” section) (Fig. 1). In Fig. 1, anomalies are shown on a percentage scale from 0 to 200, with 100 (%) representing the mean value of the entire period. The figure shows that the fluctuations in simulated and estimated biomass production are well matched with each other (Pearson’s correlation coefficients are 0.86 for the Sahelian zone and 0.64 for the Sudanian zone), showing they respond similarly to the climatic drivers. It was also noted that there were some differences between the absolute DM production derived by the two methods, such that the remotely sensed data had higher DM production than the model (~ 50%). Also a comparison of remotely sensed and ground based multi-year measurements of DM production in Sahelian and Sudanian regions of Niger and Senegal resulted in rather low R^2^ values (< 0.2–0.3)^38^. Differences between modelled and remotely sensed biomass production may be due to several reasons, including uncertainties in modelling (e.g. uncertainties in fractional cover of land-cover classes, specific site conditions regarding vegetation and soil properties, i.e. share of vegetation types (grass/ trees) and dominance of tree versus grass cover, and soil parameters), or in remote sensing estimations (e.g. transformation errors originating from different data sources, inaccurate regression models). For example, from 1988 to 2014 estimated biomass production was based on SPOT-vegetation 4 & 5 observations while afterwards Proba-V satellite observations were used. Also, overestimation of biomass production due to conversion of NDVI (Normalized Difference Vegetation Index) data into biomass (e.g. in BioHydroGenerator module, https://github.com/ACF-WARO/BioHydroGenerator) has been previously being reported^39^. Additionally, another uncertainty associated with the remotely sensed data could be due to the scarce vegetation cover in semi-arid area, where the signals are strongly influenced by the soil background^40^.

### Trend in supplied energy (S) during the historical period (1981–2020)

**Figure 2:**
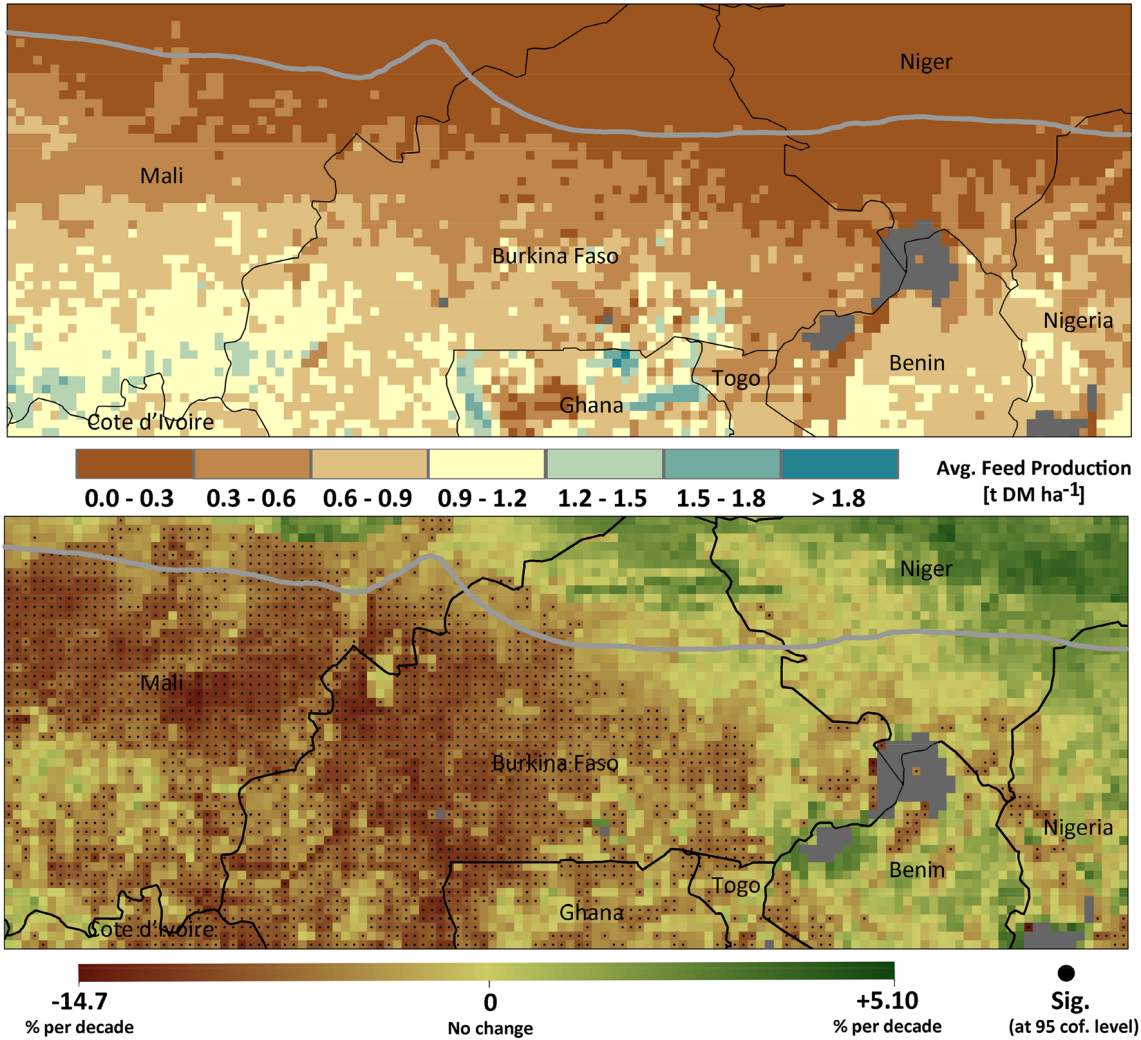
Average feed production and trend in energy supplied from the main feed resources (grass, crop residue, and brows) for livestock during the historical period of 1981 to 2020. Figures were generated using ArcGIS 10.8.1 (https://www.esri.com/en-us/arcgis/products/arcgis-pro/overview).

According to the results from modelling, the pattern of the energy supplied from the major feed resources available (herbaceous grass, crop residues (from maize, millet, sorghum, groundnut, and browse) appeared to be strongly influenced by climate variations. In Fig. 2, the mean feed production across the region and the trend in supplied energy during the historical period are shown. The simulated long-term average feed production over the whole area is ~ 0.5 tonnes ha^−1^, with a meanof ~ 0.1 tonnes ha^−1^ in the Sahelian and ~ 0.6 tonnes ha^−1^ in the Sudanian zone, respectively. For those grids predominantly covered with extensive pastoral lands, natural pasture, crop residues, and browsing provided ~ 70.0, ~ 28.5, and ~ 1.5% respectively of the total feed. In contrast, grids dominated by mixed crop-livestock systems, indicated a considerably higher dependence on crop residues, reflected by a contribution of ~ 53.0, ~ 46.0, and ~ 1.0%, for natural pasture, crop residues, and browsing, respectively.

In the great majority of the study area (~ 94%), mostly belonging to the Sudanian zone, there was decreased biomass production (~ 10.0% per decade). By contrast, for the Sahelian zone, biomass production and thus energy supply to livestock increased by about ~ 3.4% per decade (with the maximum increase of ~ 5.1% per decade). This is consistent with field measurements on herbaceous mass and satellite observations for the study area^41^, which estimate that the total vegetation production from the woody and herbaceous plants has increased by ~ 6.0–20.0% for the period 1987–2016. The observed increases in productivity for the Sahelian zone are closely related to increases in annual precipitation (trend: ~  + 0.7 mm yr^−1^), which have particularly altered early and late precipitation occurrence in the wet season (indicating an extension of the growing period). However, on average, at the regional scale, a decrease of the total energy supply prevails (on average, ~ 9.0% per decade), with a decreasing trend being statistically significant (*P* < 0.05) for about 40.0% of the study area.

### Trends in demand for energy for livestock during the historical period (1981–2020)

**Figure 3:**
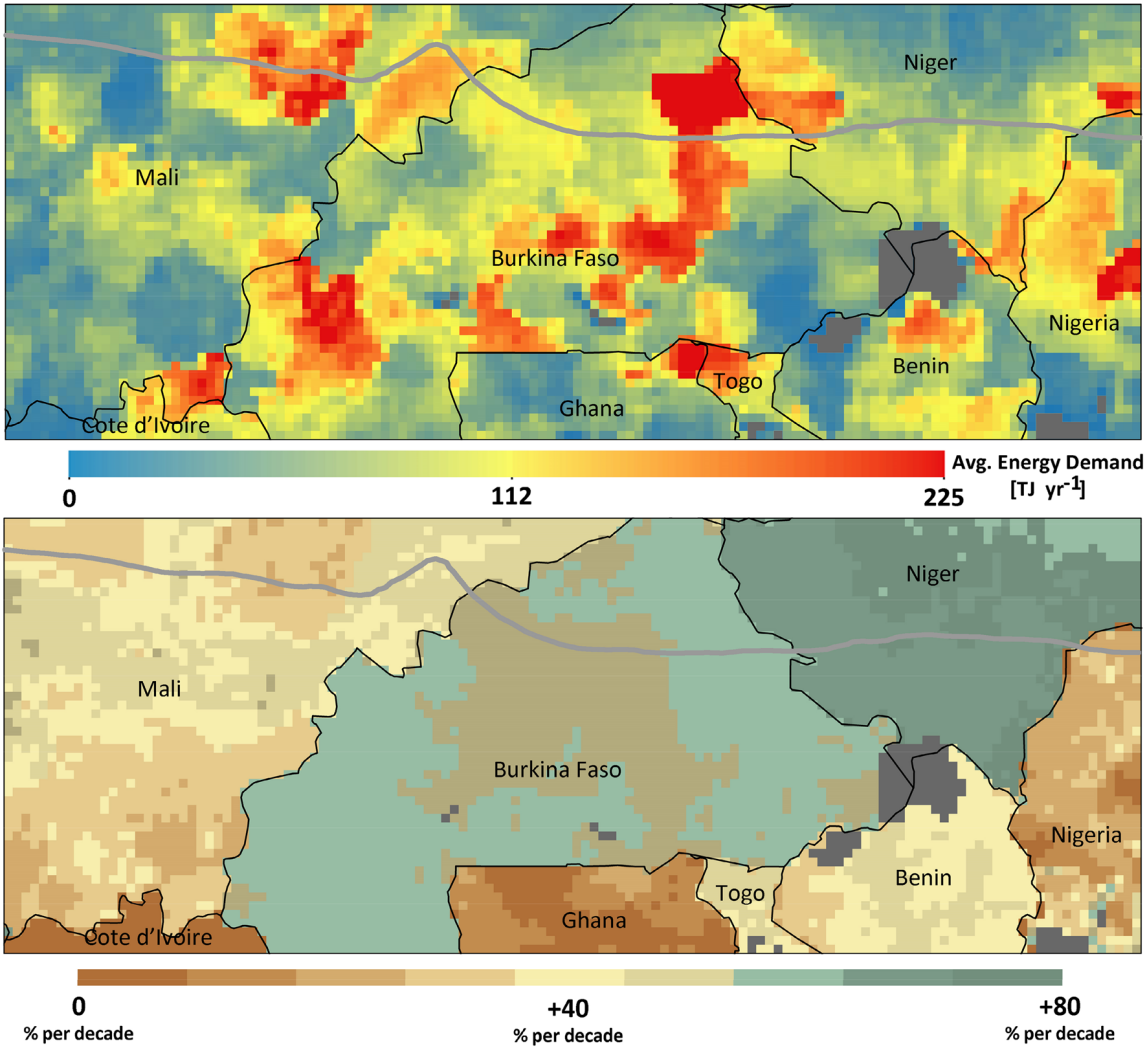
Average annual livestock energy demand (Terajoule (TJ) ME yr^−1^) and its trend during the historical period of 1981 to 2020. Figures were generated using ArcGIS 10.8.1 (https://www.esri.com/en-us/arcgis/products/arcgis-pro/overview).

The growth in regional livestock population and, thus for feed, was mainly driven by increasing local and regional demands for animal sourced proteins. This trend for increasing feed, and, thus, energy demand is reflected in Fig. 3, though due to the underlying national livestock census data differences between countries become obvious, which might not fully have reflected regional realities. According to our results, the long-term average energy demand over the whole area was ~ 40 TJ ME yr^−1^, ranging from ~ 34 TJ ME yr^−1^ in the Sahelian to ~ 41 TJ ME yr^−1^ in the Sudanian zone. Furthermore, on average, the energy demand for livestock production has increased by 43% and 35% per decade in the Sahelian and Sudanian zones, respectively. For the entire area, the demand has increased by ~ 37% per decade during the period of 1981–2020.

### Livestock carrying capacity over the historical period of 1981–2020

**Figure 4:**
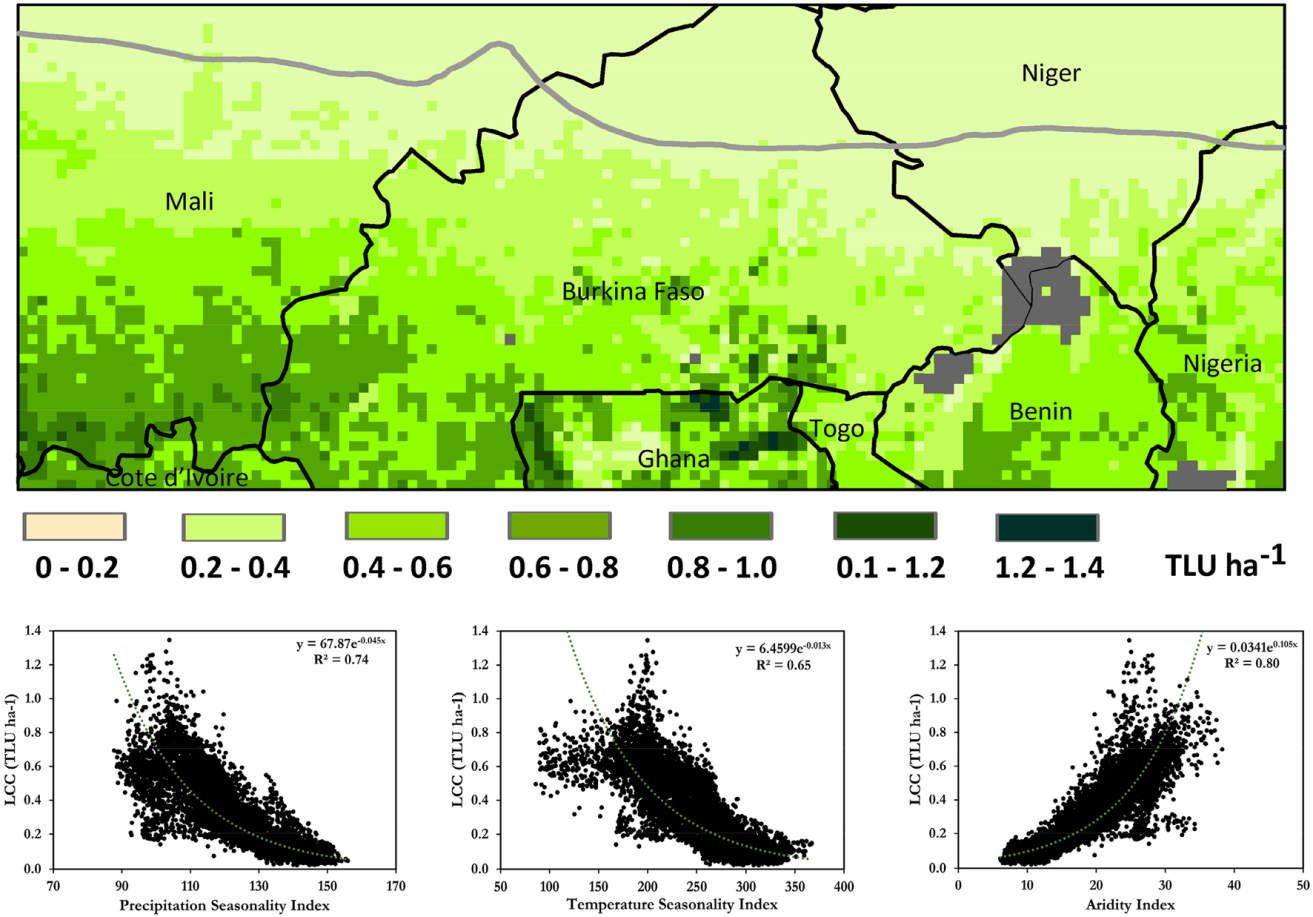
Average LCC over the historical period of 1981–2020. The lower panels show results of nonlinear regression analysis between regional bio-climatic variables and LCC. Figures were generated using ArcGIS 10.8.1 (https://www.esri.com/en-us/arcgis/products/arcgis-pro/overview) and Microsoft Excel 2016 (https://www.microsoft.com/de-de/microsoft-365/excel).

According to the obtained results, the energy demand of each TLU was calculated to be on average ~ 9400 MJ ME TLU^−1^ yr^−1^, which is within range of the general consumption rate of 1.5–2.0% of the body weight recommended by FAO^6^. Furthermore, calculating the average LCC for the entire period indicated that it ranged from 0 to 1.4 TLU ha^−1^ with an average value of 0.35 TLU ha^−1^ for the entire region (Fig. 4). However, our analysis shows that the LCC decreased from 0.58 TLU ha^−1^ in 1981 to 0.28 TLU ha^−1^ at the end of the study period. Furthermore, over the different agro-ecological zones, this was calculated to be ~ 0.1 and ~ 0.4 for the Sahelian and Sudanian zones, respectively. These results are comparable with the results obtained by previous studies from field survey for Adamawa state in Nigeria^42^ (0.6 TLU ha^−1^), Yatenga and Zondoma provinces in Burkina Faso^43^ (0.1 TLU ha^−1^). The modeled maximum 1.4 TLU ha^−1^ occurs in northern Ghana, and is less than the 2.2 TLU ha^−1^ in the more productive coastal savanna zone of Ghana^44^. The results also highlight the strength of the spatial correlation between the bioclimatic variables and the LCC (precipitation seasonality (r = 0.7), temperature seasonality (r = 0.7), and aridity indices (r = 0.8)), which could provide a simple way to first estimate the LCC (Fig. 4).

### Spatial changes in supply versus demand balance during the past four decades (1981–2020)

**Figure 5:**
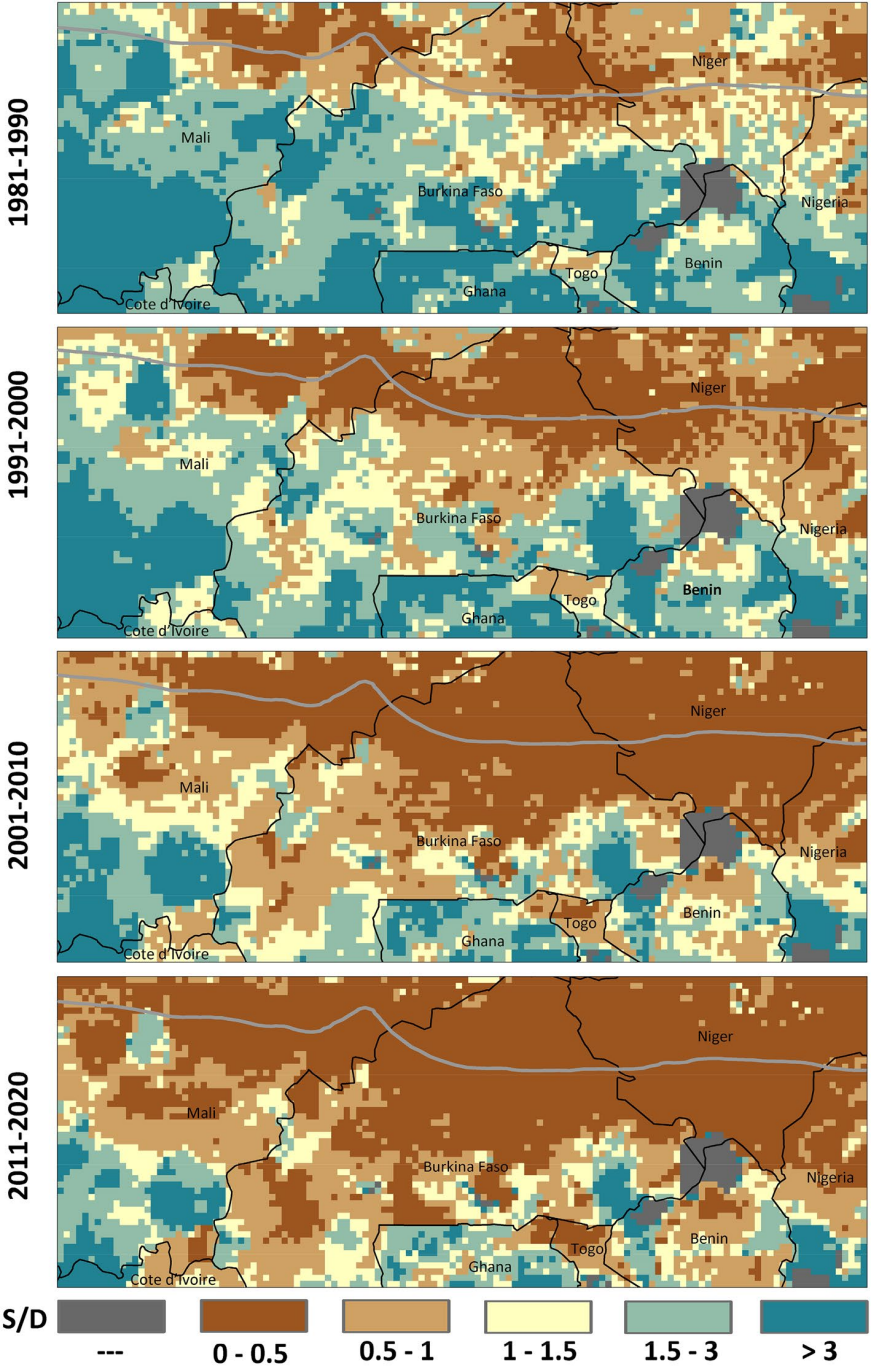
Spatial changes in supply versus demand balance (S/D) during the past four decades (1981–2020). Figures were generated using ArcGIS 10.8.1 (https://www.esri.com/en-us/arcgis/products/arcgis-pro/overview).

Calculating the total energy demand of the livestock population for each grid point in each year of the study period allowed us to assess changes in feed Supply versus feed Demand (S/D) over the past four decades (i.e. 1981–1990, 1991–2000, 2001–2010, and 2011–2020) (Fig. 5). Our analysis reveals that over the first decade of the historical period (1981–1990), only in ~ 27% of the study area (mainly in the Sahelian zone in the north) the livestock feed demand was higher than the supply. These results are also consistent with recent findings on available feed resources in Burkina Faso (Kaya and Dori regions) and Niger (Maradi and Torodi regions) using the Feed Assessment Tool (FEAST), which show shows that farmers in these regions were faced with a feed shortage due to a large livestock population^45^. Furthermore, this mismatch in S/D was addressed by purchasing feed (ranging from 13 to 23% in Dori and Torodi, respectively) and seasonal migration (ranging from 15 to 40 of the households in Kaya and Dori, respectively)^45^. However, our study also shows that over the course of the study period, more regions (42% in 1991–2000, 63% in 2001–2010, and 72% in 2011–2020), mostly in the south, have become feed deficit regions, i.e. that the feed demand of the local livestock population exceeded the supply. This is important, because as the share of the regions with more demand than the supply (S/D < 1) increases, the available mitigation strategies (like livestock mobility and buying feed from the market) become more challenging.

**Figure 6:**
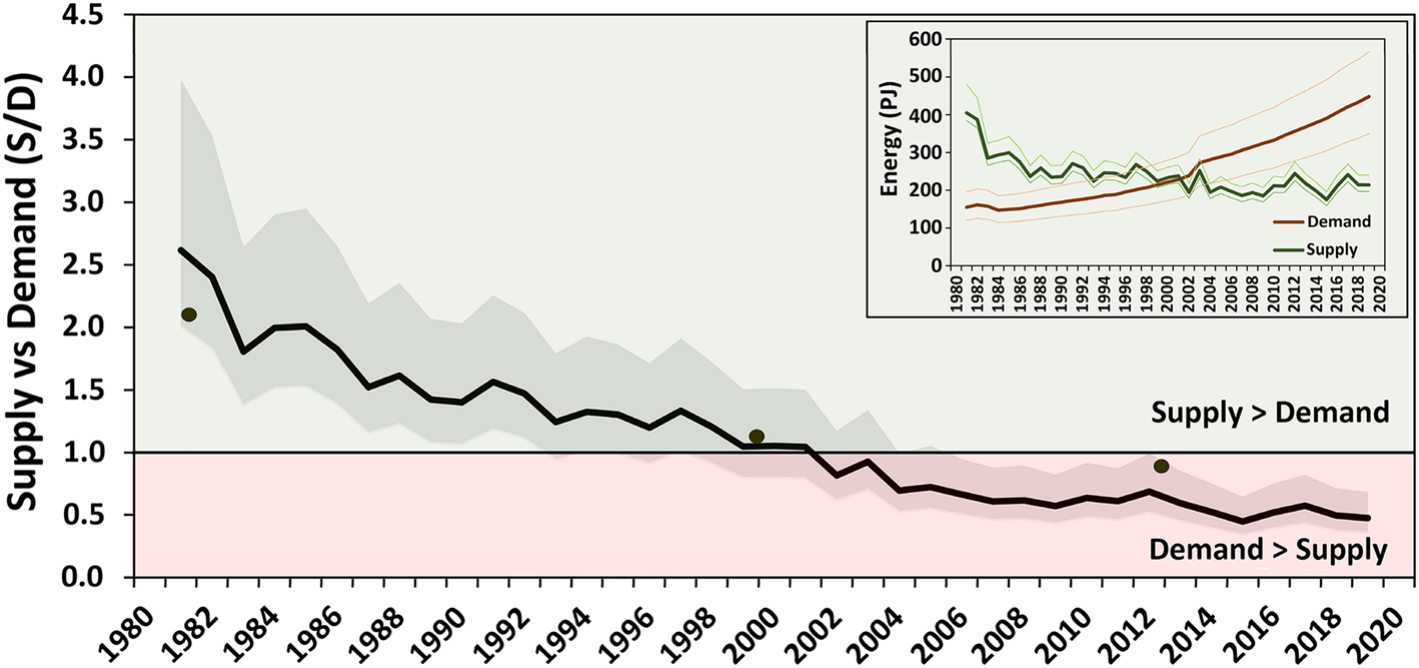
Time series and the derivation of energy supplied from the main feed resources (grass, crop residue, and brows)/energy demand of the livestock population (S/D) in the study area for the historical period of 1981 to 2020. The inset graph shows the average demand and supplied ME in Petajoule (PJ) per year. The black dots represent result if year-specific land-use maps for the years 1981, 2000, and 2013 are used. The shaded area enclosing the S/D line represents the uncertainty of simulations as originating from parametric uncertainties (Table S.1). The figure was generated using Microsoft Excel 2016 (https://www.microsoft.com/de-de/microsoft-365/excel).

To better understand the severity of this challenge and to provide a more general regional view, changes of the spatial average of S/D over the period were calculated (Fig. 6). This revealed, the S/D balance of the region has remarkably decreased during the historical period (by a rate of 0.45 per decade). Furthermore, S/D decreased from ~ 2.6 in 1981 to ~ 0.5 in 2019 and 18% of this drop was caused by reduction of supply and 82% by increase of demand (see Fig. 6 inset). The modelling indicates that, this change passed the critical level (S/D = 1) around the millennium, and since then the livestock energy demand has been greater than the feed production, meaning that the region has livestock numbers beyond its LCC.

### LCC under land-use/cover change

In order to assess how on-going changes in land-use may have affected energy availability in the study region, we calculated the S/D ratio for those years for which year-specific land-use maps were available (1981, 2000, and 2013). Comparing results for these individual years, with results using averaged and constant land use for the simulation period shows, that differences for the year 2000 are negligible, while for 1981 the supply was 12.6% higher, and for 2013 7.9% lower as compared to the mean land-use scenario (Fig. 6). The choice of land-use map affected the seasonality of the energy supply, as over years the share of cropland on total land use increased steadily. In consequence, using an average instead of a year-specific land-use map over the simulation period, resulted in an underestimation of feed production in the wet-season of 1981 by 13.5% (while dry season production was overestimated accordingly) and an underestimation of dry-season production in 2013 by 12.4%. Comparing average land use with year specific land use with regard to the balance of supply versus demand for the years 1981, 2000, and 2013 shows that the S/D ratio slightly deviate from each other, while the overall trend remains the same (average S/D versus year specific S/D for the years 1981: 2.62: 2.11; 2000: 1.05:1.15; 2013: 0.59: 0.97) (Fig. 6). For more details, also see Fig. S.4.

## Conclusions

Modelling spatio‐temporal patterns of LCC can be tackled by a variety of methods. For understanding future developments, however, it is important to determine the impacts of various drivers (environmental, socio-economical, and demographic) on the balance between energy supply and demand, which is then used to derive LCC mechanistically. With the current investigation we provide a methodology for estimating the spatio-temporal variation of supply and demand based on energy units that consider the major feed resources (natural pasture, crop residues, and browse plants) as well as a realistic livestock for the Sahelian and Sudanian zones of West Africa. Introducing a biogeochemical process-based model, LandscapeDNDC, which is especially elaborated for this task together with livestock data allowed to determine spatially resolved feed deficits and their development during the historical period of 1981–2020. From this, it can be concluded that a strong positive trend (i.e., an increase) originates from the increasing energy demand for livestock (~ 37% increase per decade) as well as a decrease in biomass production (~ 9% decrease per decade). Since the decreases in production can be related to climate conditions, i.e. changes in amount on length of rainfall period, the trend can be expected to continue with further climatic changes. The increasing expansion of deficit regions (S/D < 0) is alarming as it suggests that most of the study area already requires supplementing livestock with external feed resources, especially during the dry season. Ignoring the increasing imbalance between feed supply and demand of a growing livestock population may result in severe land degradation, specifically as opportunities for transhumance movements are shrinking and as transhumance and changes in land use/tenure (e.g. cropland expansion) are already fueling local regional conflicts between pastoralists and farming communities. Thus, the pressure on biomass resources are increasing, resulting in unsustainable use of the feed resources, a degradation of natural vegetation, overuse of crop residues (which results in soil C depletion of arable land, etc. As shown in Fig. S.5, we found a striking relationship between Supply (S)/Demand (D) for livestock feed and current estimates of degraded lands in the region indicating that with a decreasing S/D ratio land degradation increases.

On the other hand, the Sahelian and Sudainan zones of West Africa have already experienced escalating levels of violence between pastoralists and agriculturalists (crop farmers) over the last decade^46^. Although it is difficult to establish one major explanatory factor for these conflicts, it is apparent that access to land, fodder, water, and other natural resources act in tandem to perpetuate the conflicts^47^. As shown in this study, climate change in the region, together with changing demographic conditions and increases in herd sizes have turned more regions into feed deficit areas (S/D < 1) which could potentially fuel local conflicts.

Since it’s being projected that the human population^48^ and their consumption patterns^49^ will continue to change and, due to high dependency of rural populations on livestock, these changes will cause a further increase in demands for livestock products. Our results indicate that a sustainable supply will not be possible with the current level of feed production. In addition, it is likely that further land use change will happen^50^ or/and intensified management practices will be implemented, which will have an effect on LCC. This is particularly threatening since climate projections for West Africa done within the CMIP5 (The fifth phase of the Coupled Model Intercomparison Project) indicate that climate extremes will very likely increase in frequency and intensity^51,52^. Considering current state as well as future developments, our study calls for both the determination of climate-smart management options^53^, and the development of strategies to deal with the upcoming feed shortage in the area, e.g. by using alternative feeding resources, or searching for other technological interventions^54^. From the demand perspective, it would be beneficial to reflect on the prospects of reduced herd sizes, keeping less but more productive livestock, as a promising way to fight land degradation, while maintaining livestock productivity.

This study also points to a number of uncertainties which should be addressed in future studies. Firstly, a reduction of feed availability may potentially lead to expansion of livestock mobility, nomadic and semi-settled agro-pastoralists (transhumant), which are key identifying features of pastoralism in the study area^55^. However, since there are many diverse factors (e.g. political and economic barriers) controlling the livestock mobility, we only focused on modelling the self- sufficiency of livestock production in a given area by calculating the S/D ratio, thereby assuming a sustainable grazing intensity. We implicitly assumed that changes in the S/D ratio may also affect traditional mitigation strategies. Secondly, changes in land-use and -management are the two most important factors affecting feed production in West Africa. These management changes need to be modeled in a systematic manner in further investigations on LCC as e.g. forage intercropping systems have the potential to provide a better utilization of natural resources. However, as information on such systems is missing at scale of our study, we did not include such systems in our modelling framework.

Using our model approach, either different prescribed scenarios of land use or management strategies can be investigated by their impact on LCC, or adaptation strategies might be implemented in order to induce specific management options (e.g. cropping a different plant on given location, soil-fertility management, grazing pressure) as a dynamic response to variations in LCC. Climate change scenario estimates need explicit consideration, which is only possible if supply functions depend on biomass development as we have demonstrated with the LandscapeDNDC model. Finally, larger areas (as for example the whole SSA) need to be represented to consider exchange processes between regions (e.g. livestock mobility). Based on such investigations, an early warning system could be established based on the supply- demand balance as a support for decisions directed to sustainable management and sufficient feed supply.

## Material and methods

The methodology and the main steps followed in this study are illustrated in Fig. S.1 and described below.

### Study area

**Figure 7:**
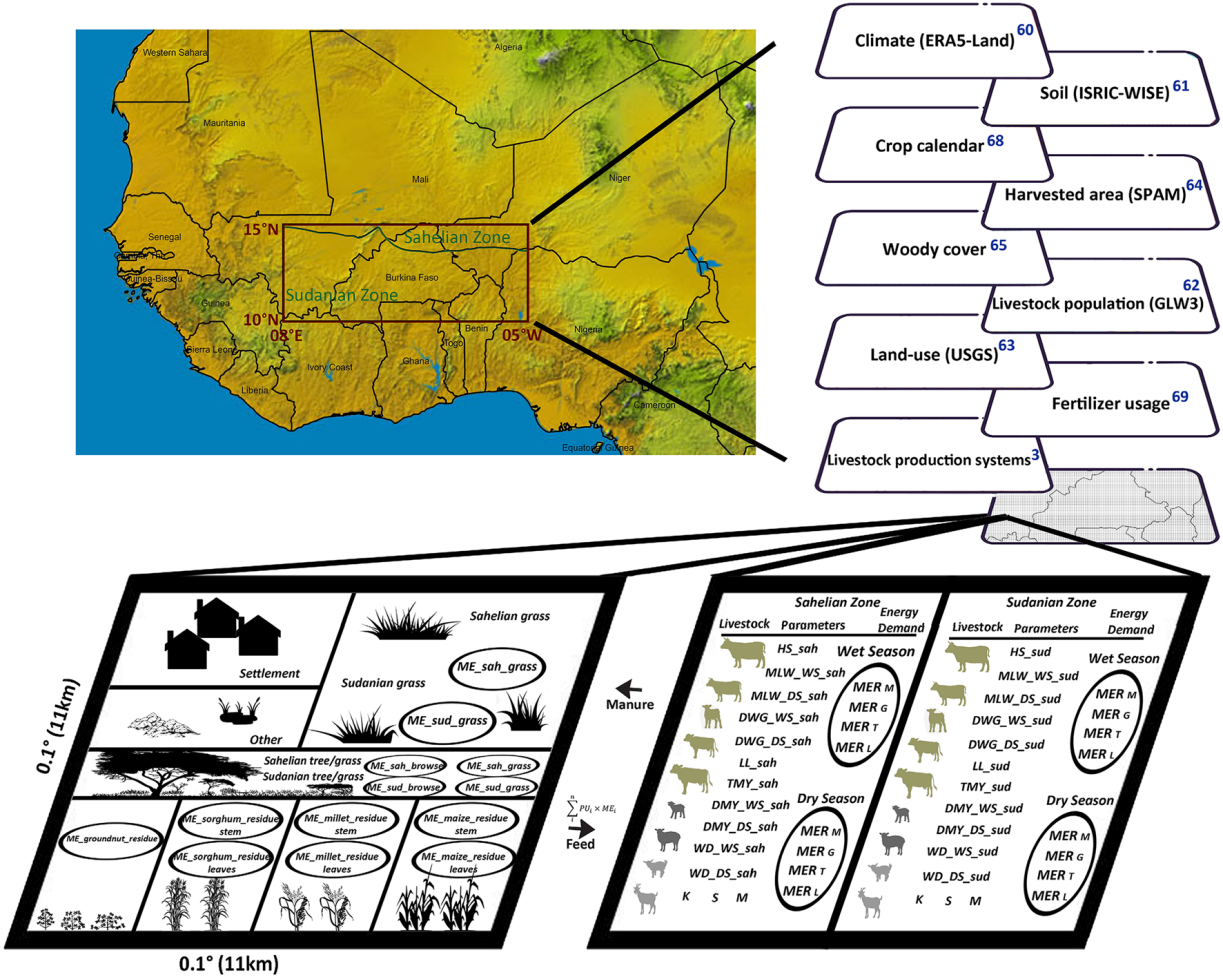
Study area, datasets used, and overview of the methodology employed for modeling energy supply and demand for a grid cell (please refer to Table S.1 in Supplementary Information for a description of each abbreviation used in this figure). Figures were generated using ArcGIS 10.8.1 (https://www.esri.com/en-us/arcgis/products/arcgis-pro/overview) and Microsoft PowerPoint 2016 (https://www.microsoft.com/de-de/microsoft-365/powerpoint).

The study area in this research, which is linked to the simulation domain of the UPSCALERS project (https://wascal.org/upscalers/), covers a portion of West Africa between a latitude of 10° and 15° N and a longitude of 08°E and 10°W, with an area of about 786,500 km^2^ (Fig. 7). The study area of the project was chosen as representative of the Sahelian and Sudanian agro-ecological zones which is where the ecosystem has been changing rapidly over the past decades. The domain is divided into 6500 grid cells of size 0.1° × 0.1° to match the resolution of the climate data (corresponds to approximately 11 × 11 km in the tropics). The northern part of the domain is located in the Sahelian belt of West Africa (~ 12% of the study area) where the mean annual precipitation during the period of 1981–2020 is ~ 245 mm/year (~ 154–392 mm/year) and the annual mean temperature is ~ 29 °C (~ 28.2–29.6 °C). The southern part of the study area is located within the Sudanian zone (~ 78% of the study area) with mean annual precipitation (1981–2020) of ~ 670 mm/year (~ 514–830 mm/year) and an annual mean temperature of ~ 28 °C (~ 27.4–28.8 °C), enabling higher vegetation productivity^56^. The wet season in the Sahelian zone of the study area lasts for only three to four months (begins in June and ends in September); in the Sudanian zone the wet season lasts for approximately five to nine months (mainly between the months of May and October). According to a high-resolution African population dataset^57^, the human population of the study area was about 38.6 million during the years 2011–2020 (Fig. S.2). Most of the people in the study area engage in crop farming and livestock husbandry activities to sustain their livelihood. The dominant livestock production systems in the area are extensive low-input pastoral systems and mixed crop-livestock systems, and the main feed resources are natural pastures, crop residues, and browsing of trees and shrubs^45,58,59^.

### Datasets used

Several global datasets have been used to model feed supply and livestock demand in the study domain, and these are listed in Fig. 7 and described below.

#### Climate dataset

The climatic parameters required for this study, including minimum and maximum air temperature (°C), precipitation (mm), wind speed (m/s), relative humidity (%), and solar radiation (W/m^2^) were obtained from the ERA5-Land offline land surface re-run of ECMWF’s (European Centre for Medium Range Weather Forecasts) latest climate product^60^.

#### Soil dataset

All soil information that is required by the applied model (i.e., pH, bulk density (kg m-3), organic C and N content (kg kg^−1^), soil texture (i.e. clay, silt, and sand content), and soil hydrological parameters (field capacity, wilting point (mm m^−3^)) were derived from the ISRIC-WISE (International Soil Reference and Information Centre-World Inventory of Soil Emission Potentials) dataset^61^.

#### Livestock population dataset

The Gridded Livestock of the World version 3 (GLW3) database^62^ (available at http://www.fao.org/livestock-systems/global-distributions/en/) provides the livestock number (cattle, sheep and goats) in each grid cell for the reference year of 2010 (with a spatial resolution of 5 arc-minutes). In the next step, for the time period of the study (i.e. 1981–2020), this dataset had been adjusted such the absolute livestock population matches official FAO’s sub-national estimates^1^. It should be also noted that, since there was no measure for the temporal development of spatial livestock distribution, we assumed that the relative livestock distribution across the historical period was the same as the GLW3 database indicates for the year 2010.

#### Livestock production system dataset

For the livestock production systems in the studied domain, a global dataset on dominant production systems (i.e. extensive pastoral systems and mixed crop-livestock systems) was used^3^.

#### Land use/cover dataset

Spatiotemporal land use/cover characteristics were derived from three raster datasets (resolution: 2 km) provided by the Comité permanent Inter-Etats de Lutte contre la Sécheresse dans le Sahel (CILSS)^63^ by counting the number of pixels of each land-use type within the 0.1° × 0.1° grid cells. These datasets provide the spatial distribution of different land-use classes (aggregated for this study to grass-, crop-, tree-dominated area, settlement area, and others) for the years 1975, 2000 and 2013. In order to determine representative land-use characteristics for the study period (1981–2020), we averaged the information across the three available points in time. However, besides results for the average land-use, we also show results for the feed supply/ demand ratio for the individual years 1981, 2000, and 2013 in Fig. 6, with land-use for 1981 being approximated by data from 1975. The percentage cover of each land use/cover category for these three years as well as the average for the entire area are shown in Fig. S.3.

#### Harvested area

The SPAM (Spatial Production Allocation Model) harvest area dataset^64^ was used to provide an estimation for the planted area of four studied crops (i.e. maize, millet, sorghum, and groundnut) in each grid cell. This dataset is a snapshot for the year 2010.

#### Woody cover

In this study, the woody cover map of the region^65^, reflecting the situation around 2016, was used for calculating the number of trees in each grid cell. These calculations were based on the Diameter at Breast Height (DBH) information for the dominant tree species in each agro-ecological zone to calculate the tree biomass^66,67^.

#### Crop calendar dataset

Crop planting and harvesting dates for maize, millet, sorghum, and groundnut were extracted from a global dataset^68^.

#### Fertilizer usage dataset

Fertilizer application values for different crops were taken from a global dataset, which gives us an overview of estimated fertilizer application rates in the area for the year 2000^69^.

#### ACF biomass production dataset

Previous attempts to estimate the biomass production in the region have mostly used remote sensing. In order to compare the model's outputs with the previously available data, we have checked our results against the biomass production anomaly produced within the ACF program. This remotely sensed dataset, which starts from the year 1988 and covers the majority of our study region, has been produced by the information from the European Space Agency (ESA) program Proba-V on basis of remotely sensed information by the SPOT 4 & 5 satellites^70^.

### The LandscapeDNDC model

In a preceding study, the process-based biogeochemical model LandscapeDNDC was parameterized and tested with regard to the prediction of biomass production in Sahelian and Sudanian agro-ecological zones in West Africa^37^. LandscapeDNDC includes various models for simulation of carbon, nitrogen, energy, and water transport along the 1-dimensional vertical soil–plant domain of different ecosystems^71^. The version of LandscapeDNDC used for this study was 1.30.4 (ref. 9953) and is available online at the Radar4KIT database (https://doi.org/10.35097/438). The model setup that was used in this study corresponds to Rahimi et al.^37^ including the biogeochemical soil model MeTr^x^^72^, the microclimate model ECM^73^, the water cycle model of DNDC^74,75^, the physiological simulation model for grasslands and grass/woodlands PSIM^73,76^, and the physiological simulation model for agricultural crops PlaMo^x^^77,78^. Details regarding the parametrization of plant physiological properties affecting carbon, nitrogen and water exchange for West African agricultural, savanna grasslands, and savanna mixed tree-grassland sites are given in Rahimi et al.^37^. In former versions of LandscapeDNDC harvest dates had to be predefined in the description of the field management model input. Hence, depending on plant development, harvest events might have been triggered before maturity. For this study, a newly developed field management model named “FarmSystem” was used that dynamically defines harvest events as soon as maturity has been reached. In addition, also the date of fertilizer application was determined dynamically. For all crop growth simulations, the complete amount of fertilizer was applied as soon as the plant reaches 25% of the Growing Degree Days (GDD) that are needed for reaching maturity. All other management factors (e.g. fertilizer amount, planting, and their ranges) were taken from the relevant dataset (see “Datasets used” section). However, we did not consider changes in crop-residue productivity over the last 40 years in our uncertainty analysis, though due to technological improvements in breeding, disease and pest control, and mechanization, grain yields were increasing. However, if increases in grain yields are also reflected in increased crop residue production is highly uncertain. It is also important to mention that, in all simulations, no irrigation was applied. We defined one tilling event occurring one day before planting. Upon tilling, residues of crops and grasses (see Table 1, proper use column) that remained on the field were transferred to the soil and incorporated into the soil C and N pools. All the simulations used a three-year spin-up to allow the model to reach its equilibrium state, particularly regarding soil carbon and nitrogen pools. Since grazing periods and intensity as well as its distribution across the region were not known, we decided to consider energy provision from grass-dominant areas in a two-step procedure. First, we simulated grass development without accounting for grazing, and then we post-processed the LandscapeDNDC output to represent the productivity under sustainable management condition. This means that an optimum grazing management is assumed which is established with grazing intervals of 10 to 11 days, and which increases productivity of the system by 7.6%^79^.

**Table 1:** Type of Feed resources used in the current investigation together with their proper use (the maximum proportion of feed resource that can be used to keep the sustainability of the ecosystem) and total ME (MJ ME kg^−1^ DM).

Type of feed		Proper use (%)	ME (MJ kg^−1^ DM)		
			Min	Max	Avg
Forages	Sahelian (e.g. *C. biflorus*)	55^80^	5.8	6.4	6.1^44,57,90,91^
	Sudanian (e.g. *A. gayanus*)	55^80^	6.2	6.8	6.5^44,57,90,91^
Browses	Sahelian (e.g. *A. tortilis*)	38	5.8	6.2	6.0^83^
	Sudanian (e.g. *B. africana*)	38	8.0	8.2	8.1^83^
Groundnut	Stem + Leaves	100^88,89^	7.2	9.3	8.5^44,57,90^
Maize residue	Stem	10^88,89^	5.7	6.3	6.0^57,88,89^
	Leaves	76^88,89^	5.9	8.3	7.1^57,88,89^
Millet residue	Stem	10^88,89^	5.2	6.0	5.6^57,88,89^
	Leaves	76^88,89^	6.8	9.4	8.1^57,88,89^
Sorghum residue	Stem	10^88,89^	5.9	6.5	6.2^57,88,89^
	Leaves	76^88,89^	5.9	6.9	6.4^57,88,89^

### Modeling energy supply

After simulating the total biomass production from different feed categories in each grid cell with the LandscapeDNDC model, these values were converted to energy supply, which is the fraction of yield that is gained under sustainable management of feed resources, by multiplying with the total Metabolizable Energy (ME) in each kg of Dry Matter (MJ ME kg^−1^ DM). These conversion factors were collected from previous studies that analyzed organic matter content, digestibility, and metabolizable energy content of the most abundant shrubs, herbs, grasses and crop species within the target region (see Table 1). On the basis of this information, only 55% of the total biomass production would be available as an energy source for livestock due to grazing efficiency, forage losses and proper use factors (which is defined as the proportion of forage that can be used by livestock without causing deterioration)^80^ (Table 1).

Regarding the crop residue, it is important to note that common post-harvest practices in West Africa result in a remarkable decrease of the nutritive value over time (depending on storage conditions and duration), and we used a factor of 8% reduction per month after the harvest date to account for this fact^81,82^.

In the current study, beside natural pastures and crop residues, browsing of understory plants in the woodlands is also considered to be an important source of energy for livestock, especially in the late dry season^83^. According to previous researches on the average annual foliage production of browse (below 2 m) in the northern and the southern parts of the study area, the dominant woody species in the area produce around 65 and 135 kg of digestible DM ha^−1^, respectively^84–86^. However, since these shrubs and trees are also important for other usages (like fuel for cooking and heating) and need to be protected against overgrazing by livestock, we assumed that only a relatively small proportion of the total above-ground production from the dominant tree/shrub species is available for browsing (take 70% and leave 30% rule^87^ × 0.55 = 0.38).

### Modeling livestock energy demand

In this study, energy requirement was calculated for different livestock categories (i.e. cattle: bull, steer, calf, heifer, cow; sheep: young stock, mature; goat: young stock, mature) for each season (wet and dry) in each grid cell. First, the total number of cattle, sheep, and goat (see “Livestock population dataset” section) were split to different age/sex categories based on the average herd characteristics in West African Sahelian and Sudanian ecosystems, since the energy requirements vary according to specific characteristics of the category (see Table S.1). Then, all the other calculations were based on the procedure given in Goopy et al.^92^, which is basically a modified form of the equations presented in the CSIRO report, Nutrient Requirements of Domesticated Ruminants^93^. In this procedure, the total Metabolizable Energy Requirements (MER) represents the sum value of the energy required for maintenance (MER_M_), growth (MER_G_), milk production (MER_L_), and locomotion (MER_T_). In this study, following the method employed in previous studies^92,94^, the energy demand for thermoregulation and pregnancy were not included because these two components are known to be less important and more uncertain than the others. It is also important to note that, in the current study, the livestock mobility, feed transfer from surplus to deficit grids, and use of supplementary feed (concentrates) were not considered due to the assumption of testing the sustainability at grid level. It means, for a given area, where the supply is less than demand (S/D < 1), farmers rely on other options (livestock mobility, feed exchanges, and using supplementary feeds) to deal with this shortage.

In our calculations for the livestock energy demand, cattle lose ~ 12% of their bodyweight in the dry season and release energy from mobilization of their body tissue^95–97^. For small ruminants, adults lose 22% of their body weight, while average daily weight gain in immatures are 30% lower during the dry period^98^. In Table S.1, all the parameters and assumptions used for modeling livestock energy demand in Sahelian and Sudanian zones are presented. The minimum, maximum, and the average of total Metabolizable Energy Requirements (MER) for different livestock types and categories in two agro-ecological zones, which are mainly dependent on the length of the wet and dry period in each grid cell, are shown in Table 2.

**Table 2:** Minimum, maximum, and the average total metabolizable energy requirements (MER) for maintenance (MER_M_), growth (MER_G_), lactation (MER_L_), travel (MER_T_) for different livestock categories roaming either in the Sahelian or Sudanian agro-ecological zone.

Livestock			Total MER (MJ/season)											
Type	Category	Age (yr.)	Sahelian zone			Sudanian zone			Sahelian zone			Sudanian zone		
			Wet season			Wet season			Dry season			Dry season		
			Min	Max	Avg	Min	Max	Avg	Min	Max	Avg	Min	Max	Avg
Cattle	Bull	≥ 3	3760	6266	5013	8703	13,055	10,879	7733	9942	8837	3786	7573	5679
	Steer	1–3	2707	4512	3609	6558	9837	8198	5065	6512	5788	2783	5566	4175
	Calf	≤ 1	1273	2122	1698	3737	5606	4672	2881	3704	3293	1718	3435	2577
	Heifer	1–3	2258	3763	3011	5387	8081	6734	4248	5461	4855	2254	4508	3381
	Cow	≥ 3	3623	6038	4830	7420	11,130	9275	6366	8185	7276	3176	6351	4764
Sheep	Young stock	≤ 1	426	711	569	1093	1639	1366	894	1149	1021	553	1107	830
	Mature	> 1	702	1169	935	1267	1901	1584	922	1185	1053	357	714	536
Goat	Young stock	≤ 1	333	554	444	899	1349	1124	702	903	803	436	873	655
	Mature	> 1	677	1128	902	1259	1888	1573	809	1035	922	354	709	532

### Bio-climatic variables

Bioclimatic variables were derived from the precipitation and temperature values in order to check the outcomes with some more ecologically meaningful variables. To this end, three variables were calculated; temperature seasonality index, precipitation seasonality index, and the aridity index. The temperature seasonality index represents temperature variation over a given year and is defined by the standard deviation of monthly temperature averages multiply by 100 ($SD\left\{ {{\text{T}}_{avg1} , \ldots ,{\text{T}}_{avg12} } \right\} \times 100$). The precipitation seasonality index, which is also known as the coefficient of variation, is defined as $\frac{{SD\left\{ {{\text{PPT}}_{1} , \ldots ,{\text{PPT}}_{12} } \right\}}}{{1 + \mathop \sum \nolimits_{i = 1}^{12} PPT_{i} }} \times 100$.

For the Aridity Index, the De Martonne’s aridity index^99^ been applied, which is defined as ${{\frac{{\mathop \sum \nolimits_{i = 1}^{12} PPT_{i} }}{12}} \mathord{\left/ {\vphantom {{\frac{{\mathop \sum \nolimits_{i = 1}^{12} PPT_{i} }}{12}} {\left( {\frac{{\mathop \sum \nolimits_{i = 1}^{12} T_{i} }}{12} + 10} \right)}}} \right. \kern-\nulldelimiterspace} {\left( {\frac{{\mathop \sum \nolimits_{i = 1}^{12} T_{i} }}{12} + 10} \right)}}$.

### Tropical livestock unit (TLU)

In order to compare the results of the current investigation with previous estimations, the LCC (stocking rate) over the region was expressed in terms of Tropical Livestock Units (TLU), weighing 250 kg, per hectare (TLU ha^−1^). To this end, total TLU of each grid point (i) was calculated by the livestock herd structure (HS), mean live weights (MLW) of each category (j), and the total population (see “Livestock population dataset” section) as $\left( {TLU_{i} = \sum\nolimits_{j = 1}^{n} {\left( {\frac{{MLW_{j} }}{250}} \right)} \times HS_{j} \times Population} \right)$.

### Statistical analysis

The non-parametric Mann–Kendall trend test was used to test the possible long-term trends in feed supply time series for each grid cell during the 1981–2020 period. In this statistical test, the null hypothesis (H0) is that there is no trend, and the alternative hypothesis (H1) is that there exist a downward or upward trend over time^100,101^. Based on the assumed level of significance α of 0.05, it is decided whether or not H_0_ is rejected, and H_1_ is accepted. Rejecting H_0_ would give indication of existing (positive or negative) trends.

### Ethical statement

Since there is no live animal involved in this study, no animal use protocol approval was required according to the guidelines/regulations for ethical principles of Karlsruhe Institute of Technology (KIT) and the Helmholtz Association. Furthermore, all datasets used in this study are publicly available via the citation given in the manuscript.

## Supplementary Information

Supplementary Information.

## Data Availability

The data products from this investigation will be available on request from the corresponding author, J.R.
