# Supplementary material for: Beyond livestock carrying capacity in the Sahelian and Sudanian zones of West Africa
Source: Sci Rep. 2021 Nov 11;11:22094. doi: 10.1038/s41598-021-01706-4 (PMC8585949; doi:10.1038/s41598-021-01706-4)
Supplement: Supplementary file 1 — Supplementary Information. [file 41598_2021_1706_MOESM1_ESM.docx]

**Supplementary Information**

**Beyond Livestock Carrying Capacity in the Sahelian and Sudanian Zones of West Africa**

Jaber Rahimi^*1^, Edwin Haas^1^, Rüdiger Grote^1^, David Kraus^1^, Andrew Smerald^1^, Patrick Laux^1^, John Goopy^2^, Klaus Butterbach-Bahl^1,2^

1 Karlsruhe Institute of Technology (KIT), Institute of Meteorology and Climate Research, Atmospheric Environmental Research (IMK-IFU), Garmisch-Partenkirchen, Germany
2 International Livestock Research Institute (ILRI), Mazingira Centre, Nairobi, Kenya

******* *Correspondence to :* Jaber Rahimi ([Jaber.rahimi@kit.edu](mailto:Jaber.rahimi@kit.edu))

**Supplementary Table 1** Parameters and assumptions used for modelling the livestock Metabolizable Energy Requirement (MER, energy demand) in Sahelian and Sudanian zones and their corresponding references

| **Parameters** | **Cattle** | | | | | **Sheep** | | **Goat** | |
| --- | --- | --- | --- | --- | --- | --- | --- | --- | --- |
|  | **Bull (> 3 yr.)** | **Steer (1-3 yr.)** | **Calf (< 1 yr.)** | **Heifer (1-3 yr.)** | **Cow (> 3 yr.)** | **Young stock (< 1 yr.)** | **Mature (> 1 yr.)** | **Young stock (< 1 yr.)** | **Mature (> 1 yr.)** |
| **HS_Sah. (%)** | 5.4 ^1^  (5-6) | 7.3 ^1^  (6-8) | 27.6 ^1^  (24-30) | 14.5 ^1^  (12-17) | 47.3 ^1^  (45-50) | 33.0 ^2,3^  (30-36) | 67.0 ^2,3^  (60-74) | 33.0 ^2,3^  (30-36) | 67.0 ^2,3^  (60-74) |
| **HS_Sud. (%)** | 14.4 ^1^  (13-15) | 11.4 ^1^  (10-12) | 20.2 ^1^  (17-23) | 16.6 ^1^  (13-19) | 37.8 ^1^  (33-41) | 33.0 ^2,3^  (30-36) | 67.0 ^2,3^  (60-74) | 33.0 ^2,3^  (30-36) | 67.0 ^2,3^  (60-74) |
| **K ^Const.^ ^4^** | 1.30 | 1.30 | 1.30 | 1.30 | 1.30 | 1.00 | 1.00 | 1.00 | 1.00 |
| **S ^Const.4^** | 1.15 | 1.15 | 1.08 | 1.00 | 1.00 | 1.08 | 1.00 | 1.08 | 1.00 |
| **M ^Const.^** | 1.00 | 1.00 | 1.08 | 1.00 | 1.00 | 1.05 | 1.00 | 1.05 | 1.00 |
| **A (yr) ^Const.^** | 3.5 | 2.5 | 0.5 | 2.5 | 3.5 | 0.5 | 1.5 | 0.5 | 1.5 |
| **MLW_WS_Sah. (kg) ^5-14^** | 260 (255-265) | 147 (114-196) | 54 (10-87) | 142 (124-166) | 234  (227-240) | 16 (14-18) | 32  (30-34) | 12  (10-14) | 25.5  (25-26) |
| **MLW_WS_Sud. (kg) ^5-14^** | 323  (265-380) | 210  (174-256) | 84  (20-134) | 195  (152-226) | 274  (253-295) | 25  (24-26) | 35  (32-38) | 19  (17-21) | 30  (28-32) |
| **MLW_DS_Sah. (kg) ^5-14^** | 229  (224-234) | 129  (100-158) | 54  (10-87) | 125  (109-141) | 205  (198-212) | 16  (14-18) | 25  (23-27) | 12  (10-14) | 20  (18-22) |
| **MLW_DS_Sud. (kg) ^5-14^** | 284  (277-291) | 185  (153-217) | 84  (20-134) | 172  (134-210) | 241  (221-261) | 25  (24-26) | 27  (25-29) | 19  (15-23) | 23  (20-26) |
| **DWG_WS_Sah. (kg) ^Const. 5-14^** | 0.246 | 0.471 | 0.103 | 0.329 | 0.308 | 0.100 | 0.192 | 0.072 | 0.162 |
| **DWG_WS_Sud. (kg) ^Const. 5-14^** | 0.258 | 0.252 | 0.319 | 0.192 | 0.144 | 0.075 | 0.073 | 0.058 | 0.072 |
| **DWG_DS_Sah. (kg) ^Const. 5-14^** | -0.015 | -0.028 | 0.001 | -0.020 | -0.019 | 0.027 | -0.029 | 0.019 | -0.023 |
| **DWG_DS_Sud. (kg) ^Const. 5-14^** | -0.062 | -0.061 | 0.001 | -0.046 | -0.035 | 0.076 | -0.063 | 0.028 | -0.055 |
| **M/D_WS_Sah. (MJ/kg DM)** | 6.1  (5.8-6.4) | 6.1  (5.8-6.4) | 6.1  (5.8-6.4) | 6.1  (5.8-6.4) | 6.1  (5.8-6.4) | 6.1  (5.8-6.4) | 6.1  (5.8-6.4) | 6.1  (5.8-6.4) | 6.1  (5.8-6.4) |
| **M/D_WS_Sud. (MJ/kg DM)** | 6.5  (6.2-6.8) | 6.5  (6.2-6.8) | 6.5  (6.2-6.8) | 6.5  (6.2-6.8) | 6.5  (6.2-6.8) | 6.5  (6.2-6.8) | 6.5  (6.2-6.8) | 6.5  (6.2-6.8) | 6.5  (6.2-6.8) |
| **M/D_DS_Sah. (MJ/kg DM)** | 6.06  (5-7) | 6.06  (5-7) | 6.06  (5-7) | 6.06  (5-7) | 6.06  (5-7) | 6.06  (5-7) | 6.06  (5-7) | 6.06  (5-7) | 6.06  (5-7) |
| **M/D_DS_Sud. (MJ/kg DM)** | 6.27  (5-8) | 6.27  (5-8) | 6.27  (5-8) | 6.27  (5-8) | 6.27  (5-8) | 6.27  (5-8) | 6.27  (5-8) | 6.27  (5-8) | 6.27  (5-8) |
| **LL (d/yr) ^Info. 3, 10, 15-18^** | - | - | - | - | 210  (180-240) | - | 135  (120-150) | - | 240  (225-270) |
| **MDMP (lit./day) ^Info. 3, 18^** | - | - | - | - | 3.1 ^21^  (2.4-3.8) | - | 0.6  (0.5-0.8) | - | 0.6  (0.5-0.8) |
| **TMY_Sah. (lit./yr) ^3, 18^** |  |  |  |  | 500  (500-800) |  | 40  (40-50) |  | 110  (110-150) |
| **TMY_Sud. (lit/yr) ^3, 18^** |  |  |  |  | 800  (500-800) |  | 50  (40-50) |  | 150  (110-150) |
| **DMY_WS_Sah. (lit.) ^Calc. ***^** | - | - | - | - | 3.33 | - | 0.27 | - | 0.73 |
| **DMY_WS_Sud. (lit.) ^Calc. ***^** |  |  |  |  | 2.67 |  | 0.17 |  | 0.50 |
| **DMY_DS_Sah. (lit.) ^Calc.^ ^19***^** | - | - | - | - | 0.42 | - | 0.03 | - | 0.09 |
| **DMY_DS_Sud. (lit.) ^Calc.^ ^19***^** |  |  |  |  | 1.33 |  | 0.08 |  | 0.25 |
| **EC^20^** | 20 | 20 | 20 | 20 | 20 | 20 | 20 | 20 | 20 |
| **WD_WS_Sah. (km/d) ^Const. 17, 21^** | 5.5 | 5.5 | 2.5 | 5.5 | 2.5 | 2.5 | 5.5 | 2.5 | 5.5 |
| **WD_WS_Sud. (km/d) ^Const.^ ^17^** | 5.5 | 5.5 | 2.5 | 5.5 | 2.5 | 2.5 | 5.5 | 2.5 | 5.5 |
| **WD_DS_Sah. (km/d) ^Const. 17, 21^** | 5.5 | 5.5 | 5.5 | 2.5 | 5.5 | 2.5 | 5.5 | 2.5 | 5.5 |
| **WD_DS_Sud. (km/d) ^Const. 17^** | 5.5 | 5.5 | 5.5 | 2.5 | 5.5 | 2.5 | 5.5 | 2.5 | 5.5 |
| **MER_m__WS_Sah. (MJ/d) ^Calc.^** | 39.23 | 26.31 | 13.35 | 22.33 | 31.47 | 4.00 | 5.79 | 3.17 | 4.88 |
| **MER_m_ (MJ/d)_wet_sud ^Calc.^** | 45.53 | 34.01 | 18.40 | 27.97 | 35.03 | 5.49 | 6.06 | 4.55 | 5.45 |
| **MER_m__DS_Sah. (MJ/d) ^Calc.^** | 35.69 | 23.94 | 13.37 | 20.32 | 28.63 | 4.01 | 5.10 | 3.17 | 4.58 |
| **MER_m__DS_Sud. (MJ/d) ^Calc.^** | 41.67 | 31.13 | 18.54 | 25.60 | 32.07 | 5.53 | 4.21 | 4.58 | 3.78 |
| **MER_G__WS_Sah (MJ/d) ^Calc.^** | 1.72 | 3.30 | 0.72 | 2.31 | 2.16 | 0.70 | 1.35 | 0.50 | 1.13 |
| **MER_G__WS_Sud. (MJ/d) ^Calc.^** | 1.81 | 1.77 | 2.24 | 1.34 | 1.01 | 0.53 | 0.51 | 0.41 | 0.51 |
| **MER_G__DS_Sah. (MJ/d) ^Calc.^** | -0.34 | -0.65 | 0.00 | -0.45 | -0.43 | 0.19 | -0.67 | 0.13 | -0.54 |
| **MER_G__DS_Sud. (MJ/d) ^Calc.^** | -1.43 | -1.39 | 0.01 | -1.06 | -0.79 | 0.54 | -1.46 | 0.20 | -1.27 |
| **MER_T__WS_Sah. (MJ/d) ^Calc.****^** | 3.72 | 2.10 | 0.35 | 2.03 | 1.52 | 0.10 | 0.46 | 0.08 | 0.36 |
| **MER_T__WS_Sud. (MJ/d) ^Calc.****^** | 4.61 | 3.00 | 0.55 | 2.79 | 1.78 | 0.16 | 0.49 | 0.13 | 0.43 |
| **MER_T__DS_Sah. (MJ/d) ^Calc.****^** | 3.27 | 1.85 | 0.77 | 0.81 | 2.94 | 0.10 | 0.39 | 0.08 | 0.33 |
| **MER_T__DS_Sud. (MJ/d) ^Calc.****^** | 4.06 | 2.64 | 1.21 | 1.12 | 3.45 | 0.16 | 0.30 | 0.13 | 0.26 |
| **MER_L__WS_Sah. (MJ/d) ^Calc.^** | 0.00 | 0.00 | 0.00 | 0.00 | 6.28 | 0.00 | 0.50 | 0.00 | 1.38 |
| **MER_L__WS_Sud. (MJ/d) ^Calc.^** | 0.00 | 0.00 | 0.00 | 0.00 | 4.79 | 0.00 | 0.30 | 0.00 | 0.90 |
| **MER_L__DS_Sah. (MJ/d) ^Calc.^** | 0.00 | 0.00 | 0.00 | 0.00 | 0.79 | 0.00 | 0.06 | 0.00 | 0.17 |
| **MER_L__DS_Sud. (MJ/d) ^Calc.^** | 0.00 | 0.00 | 0.00 | 0.00 | 2.46 | 0.00 | 0.15 | 0.00 | 0.46 |
| **TMER _WS_Sah. (MJ/WS) ^Calc.^** | 5012.91 | 3609.38 | 1697.97 | 3010.71 | 4830.34 | 568.60 | 935.43 | 443.50 | 902.49 |
| **TMER _WS_Sud. (MJ/WS) ^Calc.^** | 11604.55 | 8744.31 | 4982.95 | 7183.06 | 9893.62 | 1456.71 | 1689.39 | 1199.08 | 1678.17 |
| **TMER_DS_Sah. (MJ/DS) ^Calc.^** | 8837.17 | 5788.34 | 3292.75 | 4854.62 | 7275.73 | 1021.41 | 1053.41 | 802.80 | 924.82 |
| **TMER_DS_Sud. (MJ/DS) ^Calc.^** | 5048.33 | 3710.74 | 2290.33 | 3005.52 | 4234.26 | 737.85 | 476.12 | 581.94 | 472.56 |
| \| - Sah.= Sahelian Zone \| \| --- \| \| - Sud. = Sudanian Zone \| \| - K = Intermediate values for *B. indicus* and *B. taurus* breeds, 1.0 for sheep and goats \| \| - S = 1 for females, 1.15 for males, and intermediate value for immature \| \| - M = 1 + (0.23× proportion of digestible energy from milk (~0.25-0.35)) \| \| - MLW = Mean Live Weight \| \| - WS = Wet Season \| \| - DS = Dry Season \| \| - DWG = Daily Weight Gain \| \| - A = Age \| \| - M/D = MJ of ME per kg dry matter \| \| - LL = Lactation Length \| \| - TMY = Total Milk Yield \| \| - MDMP = Mean Daily Milk Production \| \| - DMY = Daily Milk Yield ($DMY=\frac{({TMY}_{Season}(kg))}{Number of days in season}$) \| \| - EC = Energy Content of the tissue - WD = Walking Distance \| \| - MER_M_ = Metabolizable Energy Requirements for Maintenance (${MER}_{M}=K\times S\times M\times\frac{(0.26\times{MLW}^{0.75}(kg)\times{exp}^{\left( -0.03\times A \right)})}{\left( 0.02\times\frac{M}{D} \right)+0.5}$) \| \| - MER_G_ = Metabolizable Energy Requirements for Growth (If weight change over the period was positive then: ${MER}_{G}=\frac{({TMY}_{Season}(kg)\times0.92\times EC(energy content of the tissue=MJ/kg))}{\left( 0.043\times\frac{M}{D} \right)}$ ; if negative then ${MER}_{G}=\frac{({TMY}_{Season}\times0.92\times EC)}{\left( 0.8 \right)}$ \| \| - MER_T_ = Metabolizable Energy Requirements for Locomotion (${MER}_{T}=WD(km)\times MLW(kg)\times0.0026\left[ \frac{MJ}{KgLW}/km \right]$) \| \| - MER_L_ = Metabolizable Energy Requirements for Lactation (${MER}_{L}=\frac{(DMY(kg)\times ECM(Energy content of milk; taken as 3.054\frac{MJ}{kg}\left( CSIRO, 2007 \right)))}{\left( 0.02\times\frac{M}{D} \right)+0.04}$) \| \| - TMER = Total Metabolizable Energy Requirements _assuming average number of days of wet and dry season in Sahelian and Sudanian zones (see Calc.) - Const. = Constant value - Info. = more information - Calc. = Calculated based on the average length of wet and dry season in Sahelian (wet season=four months, dry season=eight months) and Sudanian (wet season=eight months, dry season=four months) agro-ecological zones \| \| - * On average, adults lose 12% of their bodyweight in dry season ^22-24^ \| \| - ** Adult small ruminants lose 22% of their body weight, while average daily weight gain in immatures are 30% lower during the dry period ^3^ \| \| - *** Milk production is assumed to be 80% higher in wet season \| \| - **** Mobility average of each category during wet and dry season, respectively: cattle: 22% and 45%; sheep: 34% and 53%; goat 33% and 56% ^25^ \| \| 1: Amanor (1995); 2: Otte and Chilonda (2002); 3: Wilson (1991); 4: CSIRO (2007); 5: Schlecht et al. (2019); 6: Adjibode et al. (2017); 7: Lambourne (1985); 8: Birteeb et al. (2015); 9: Gbangboche et al. (2005); 10: Rege et al. (1994); 11: Wilson (1988); 12: Simões and Gutiérrez (2018); 13: Birteeb and Ozoje (2012); 14: Joshi et al. (1957); 15:Jaitne et al. (2006); 16:Adewumi and Olorunnisomo (2009); 17:Adriansen and Nielsen (2005); 18: Le Houerou (2012); 19: Daodu and Babayemi (2008); 20: Konandreas and Anderson (1982); 21: Zampaligré (2012); 22: Dwinger et al. (1994); 23: Senock and Pieper (1990); 24: Wagenaar (1986); 25: Turner et al. (2014) \| | | | | | | | | | |


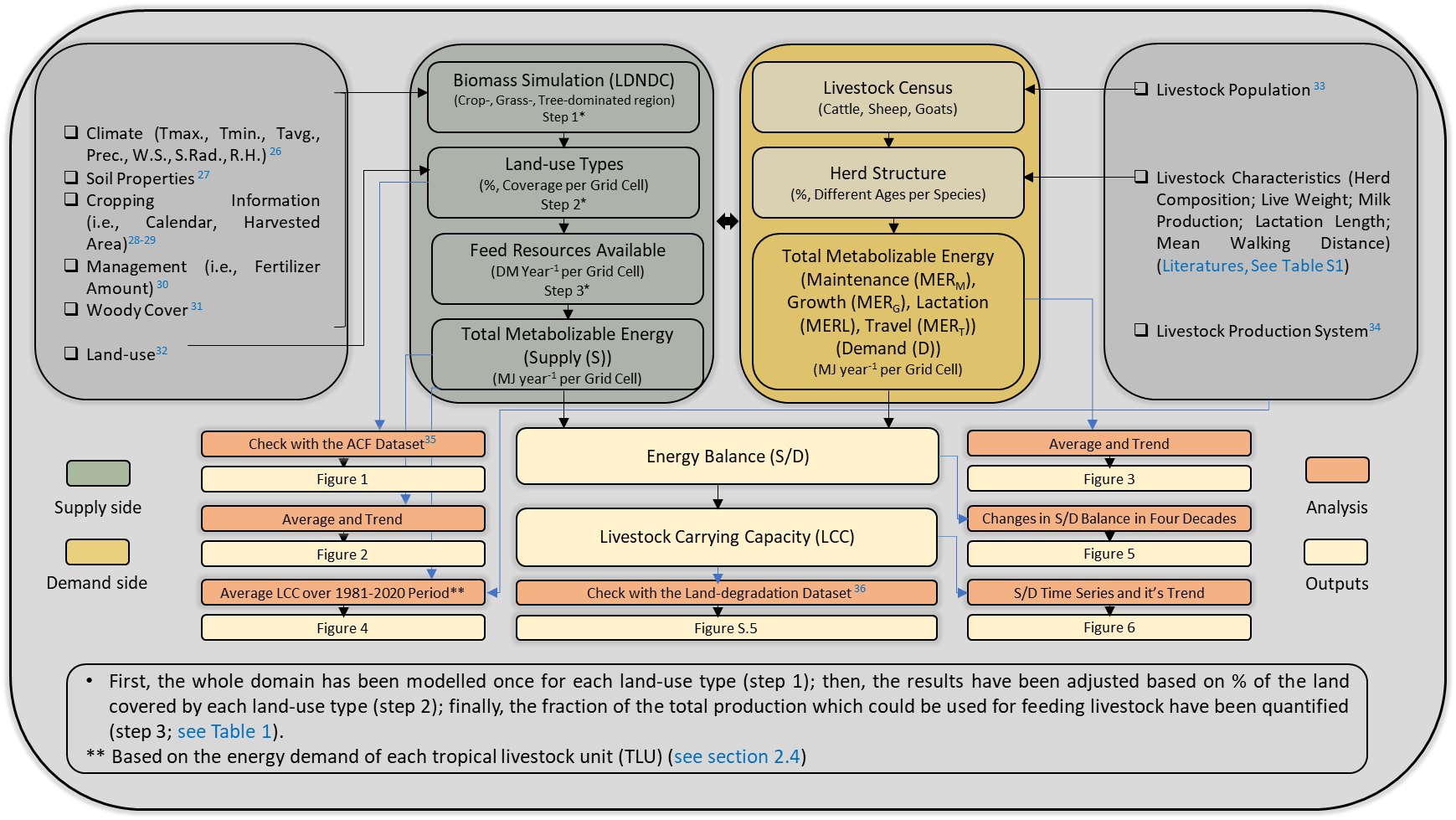


**Supplementary Figure 1** Research methodology flow chart.


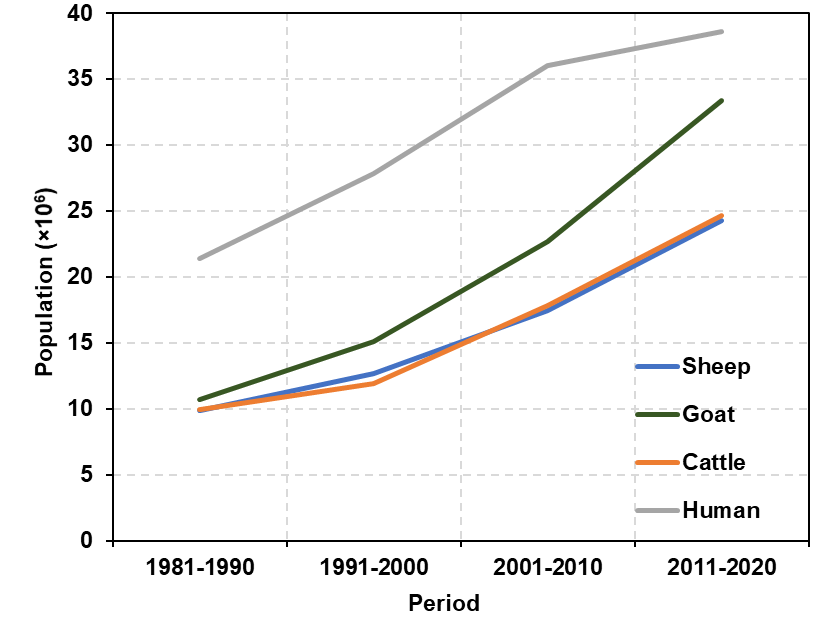


**Supplementary Figure 2** Changes in total number of livestock (cattle, sheep, and goat) and human population in the study area from 1981 – 2020. The figure was generated using Microsoft Excel 2016 (https://www.microsoft.com/de-de/microsoft-365/excel).

| 1975 | 2000 |
| --- | --- |
| 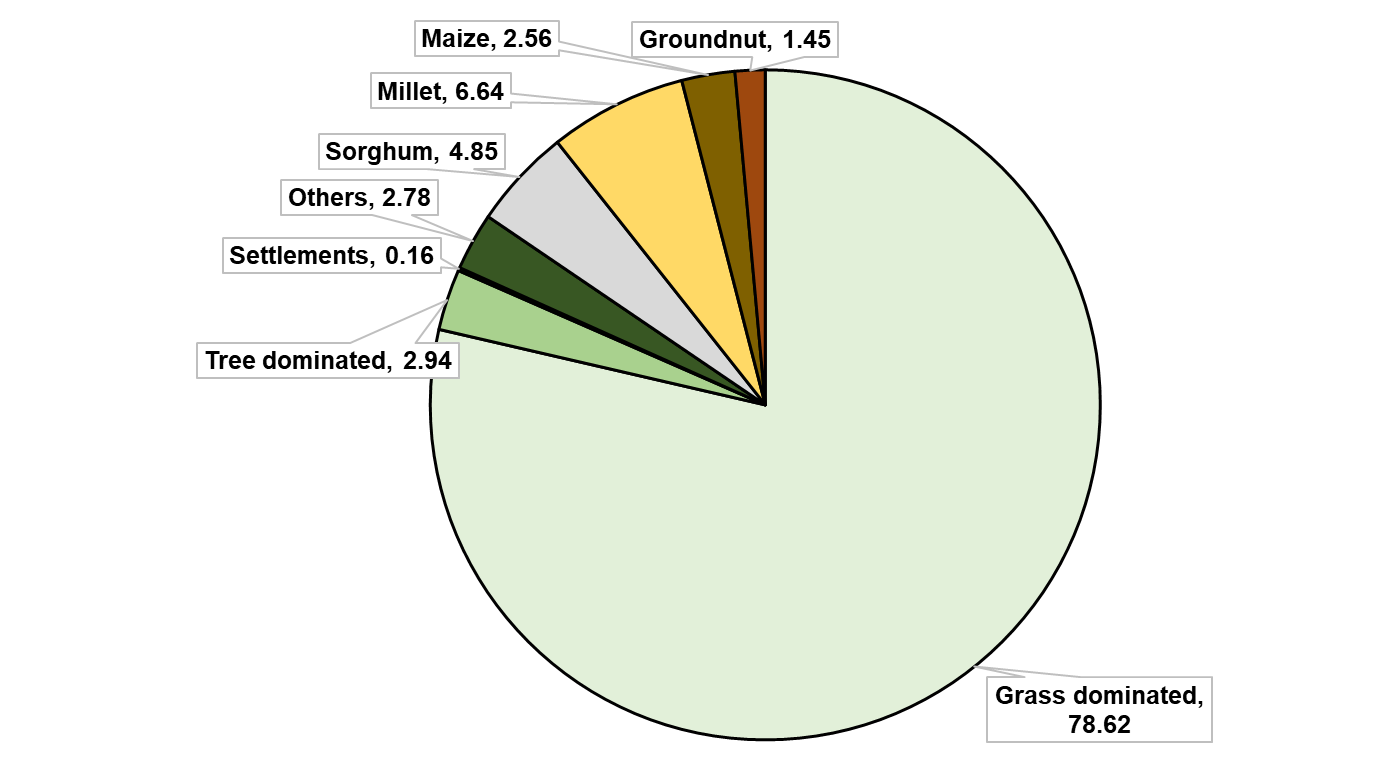 | 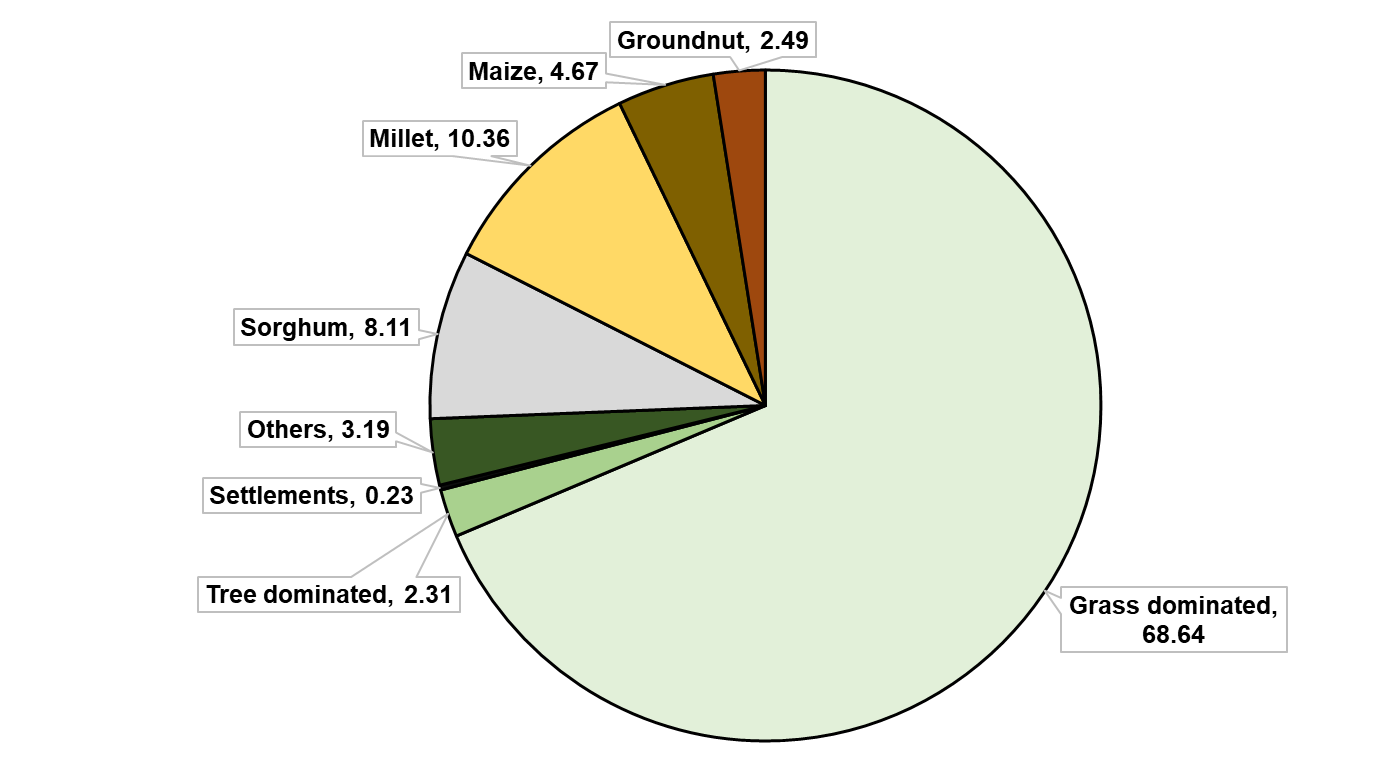 |
| 2013 | Average |
| 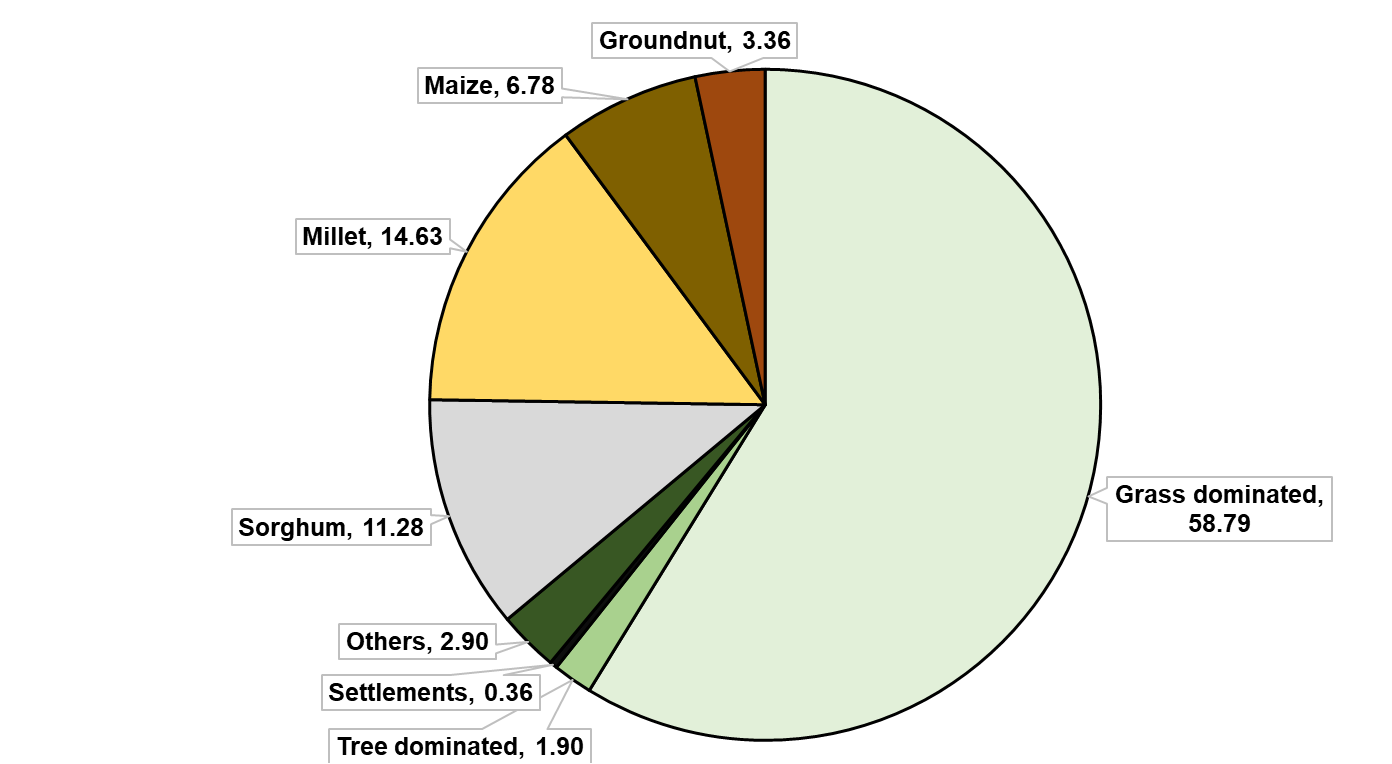 | 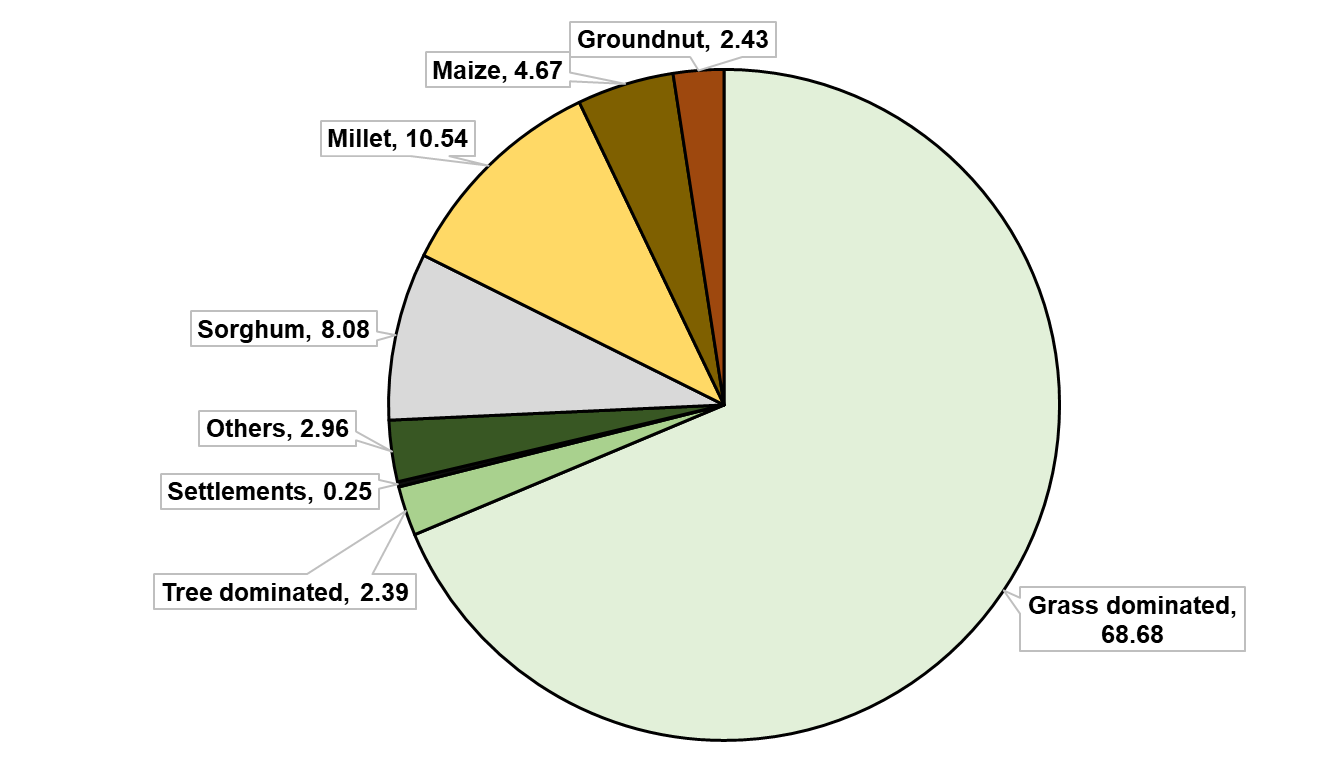 |

**Supplementary Figure 3** The land-use/cover conditions of the study in 1975, 2000, and 2013, as well as the average fractional cover of land-use/cover each category during the historical period. The figure was generated using Microsoft Excel 2016 (https://www.microsoft.com/de-de/microsoft-365/excel).

**Supplementary Figure 4** Spatial changes in supply vs. demand balance (S/D) in 1981 (approximated by the data from 1975), 2000, and 2013. Figures were generated using ArcGIS 10.8.1 (https://www.esri.com/en-us/arcgis/products/arcgis-pro/overview).

**
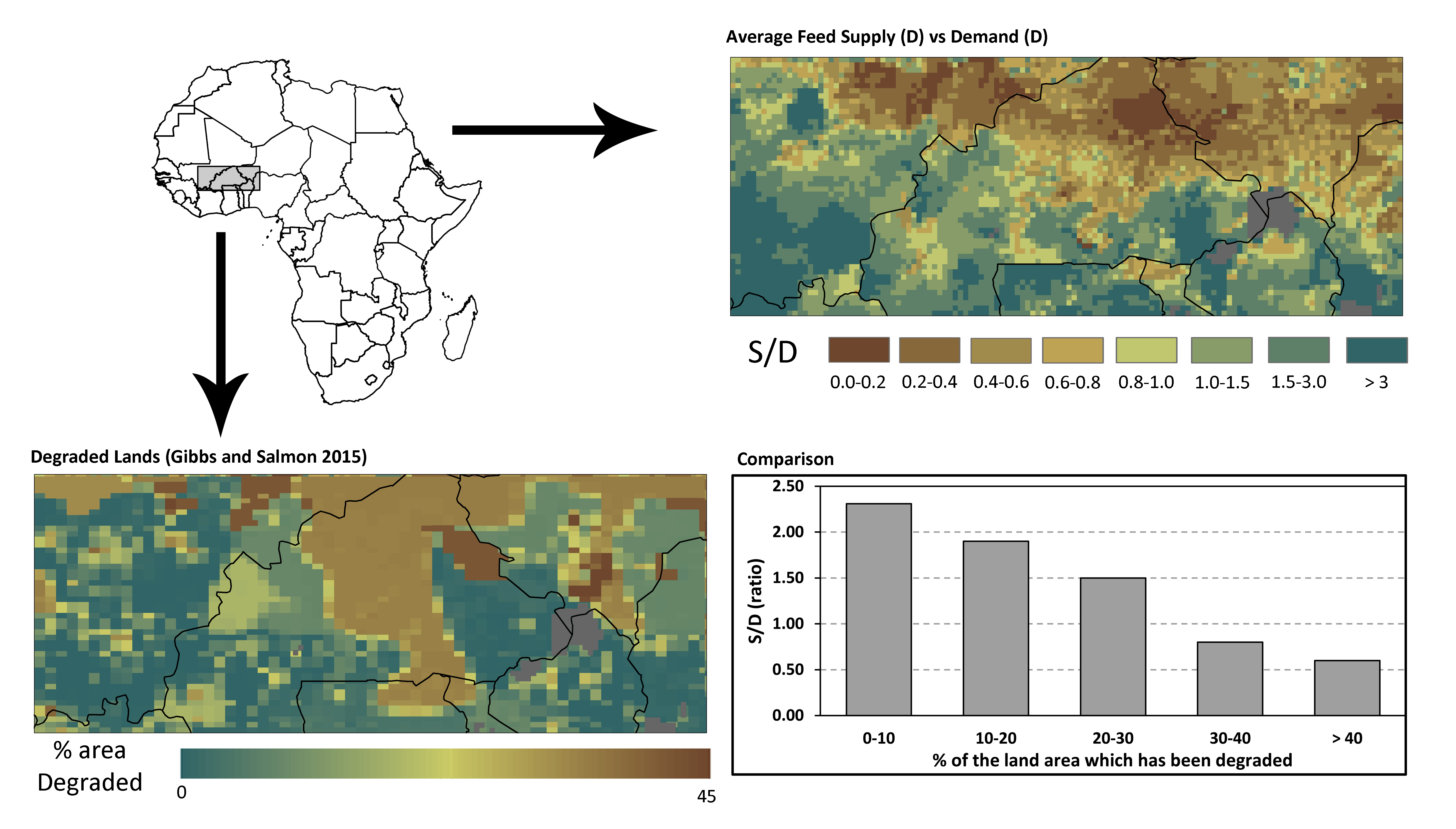
**

**Supplementary Figure 5** Comparison of regional differences in Supply (S)/Demand (D) for livestock feed (own study) and % of the land area which has been degraded as created by Gibbs and Salmon (2015)^26^. Meaning that, on average, in regions where more than 40% of the land is being degraded, the S/D is ~0.6. Figures were generated using ArcGIS 10.8.1 (https://www.esri.com/en-us/arcgis/products/arcgis-pro/overview) and Microsoft Excel 2016 (https://www.microsoft.com/de-de/microsoft-365/excel).

**References**

1. Amanor, K. S. Dynamics of herd structures and herding strategies in West Africa: A study of market integration and ecological adaptation. *Africa* 65(3), 351-394 (1995).
2. Otte, M. J. & Chilonda, P. Cattle and small ruminant production systems in sub-Saharan Africa. A systematic review. Food and Agriculture Organization of the United Nations, Rome, Italy (2002).
3. Wilson, R. T. Small ruminant production and the small ruminant genetic resource in tropical Africa. Production and Health Paper No. 88, FAO, Rome. (1991).
4. CSIRO. (2007). Nutrient requirements of domesticated ruminants. CSIRO publishing.
5. Schlecht, E., Plagemann, J., Mpouam, S. E., Sanon, H. O., Sangaré, M. & Roessler, R. Input and output of nutrients and energy in urban and peri-urban livestock holdings of Ouagadougou, Burkina Faso. *Nutrient Cycling in Agroecosystems* 115(2), 201-230 (2019).
6. Adjibode, G., Tougan, U. P., Daouda, I. H., Mensah, G. A., Youssao, A. K. I., Hanzen, C., Thewis, A. & Koutinhouin, G. B. Factors affecting reproduction and growth performances in West African Dwarf sheep in sub-Saharan Africa. *International Journal of Agronomy and Agricultural Research* 11(1), 60-68 (2017).
7. Lambourne, L. J. Research in goat productivity in tropical Africa. in: Goat Production and Research in the Tropics, (Ed: J. W. Copland). Aust. Centre for Int. Agric. Res., Canberra, 53-65 (1985).
8. Birteeb, P. T., Danquah, B. A. & Salifu, A. S. Growth performance of West African Dwarf Goats reared in the transitional zone of Ghana. *Asian Journal of Animal Sciences* 9(6), 370-378 (2015).
9. Gbangboche, A., Abiola, F. A. & Leroy, P. Comparison of West African Dwarf (WAD) sheep and F1 crossed West African Long Legged (WALL) rams with WAD ewes in Benin: Growth and Survival traits. *The Global Food & Product Chain—Dynamics, Innovations, Conflicts, Strategies* Deutscher Tropentag 2005 (2005).
10. Rege, J. E. O., Aboagye, G. S. & Tawah, C. L. Identification and characterization of West African Shorthorn cattle. *World Animal Review* 78(1), 1-12 (1994).
11. Wilson, R. T. The productivity of Sahel goats and sheep under transhumant management in Northern Burkina Faso. *Bulletin of Animal Health and Production in Africa* 36, 348-355 (1988).
12. Simões, J. & Gutiérrez, C. (Eds.). Sustainable Goat Production in Adverse Environments: Volume I: Welfare, Health and Breeding. Springer (2018).
13. Birteeb, P. T. & Ozoje, M. O. Prediction of live body weight from linear body measurements of West African long-legged and West African dwarf sheep in northern Ghana. *Online Journal of Animal and Feed Research* 2(5), 427-434 (2012).
14. Joshi, N. R., Mclaughlin, E. A. & Phillips, R. W. Types and breeds of African cattle. Types and breeds of African cattle. FAO, ROME, 306 p. (1957).
15. Jaitner, J., Njie, M., Corr, N. & Dempfle, L. Milk production of West African Dwarf goats in the Gambia. *Tropical Animal Health and Production* 38(3), 261-266 (2006).
16. Adewumi, O. O. & Olorunnisomo, A. O. Milk yield and milk composition of West African dwarf, Yankasa and crossbred sheep in southwest of Nigeria. *Livestock Research for Rural Development* 21(3), 1-8 (2009).
17. Adriansen, H. K. & Nielsen, T. T. The geography of pastoral mobility: A spatio-temporal analysis of GPS data from Sahelian Senegal. *GeoJournal* 64(3), 177-188 (2005).
18. Le Houerou, H. N. The grazing land ecosystems of the African Sahel (Vol. 75). Springer Science & Business Media. (2012).
19. Daodu, M. O. & Babayemi, O. J. Milk production capacity of dairy cattle under limited resources and distribution pattern in peri-urban area of southwest Nigeria. In Competition for resources in a changing world: New drive for rural development. *Conference of the International Research on Fodd Security, Natural Resource Management and Rural Development*, Tropentag, 7th-9th October. (2008).
20. Konandreas, P. A. & Anderson, F. M. Cattle herd dynamics: an integer and stochastic model for evaluating production alternatives. *ILRI (aka ILCA and ILRAD)* (1982).
21. Zampaligré, N. The role of ligneous vegetation for livestock nutrition in the sub-Sahelian and Sudanian zones of West Africa: Potential effects of climate change. *Cuvillier Verlag* (2012).
22. Dwinger, R. H., Agyemang, K., Snow, W. F., Rawlings, P., Leperre, P. & Bah, M. L. Productivity of trypanotolerant cattle kept under traditional management conditions in the Gambia. *Veterinary Quarterly* 16(2), 81-86 (1994).
23. Senock, R. S. & Pieper, R. D. Livestock Production constraints in the Sahel. *Rangelands* 12(4), 225-229 (1990).
24. Wagenaar, K. T. Productivity of transhumant Fulani cattle in the inner Niger delta of Mali (Vol. 13). ILRI (aka ILCA and ILRAD) (1986).
25. Turner, M. D., McPeak, J. G. & Ayantunde, A. The role of livestock mobility in the livelihood strategies of rural peoples in semi-arid West Africa. *Human Ecology* 42(2), 231-247 (2014).
26. ECMWF. ERA5-Land hourly data from 1981 to present, Tech. rep., ECMWF, https://doi.org/10.24381/cds.e2161bac. (2019)
27. Batjes, N. H. ISRIC-WISE Harmonized Global Soil Profile Dataset. ISRIC-World Soil Information, Wageningen (2008).
28. Yu, Q., You, L., Wood-Sichra, U., Ru, Y., Joglekar, A.K., Fritz, S., Xiong, W., Lu, M., Wu, W. & Yang, P. A cultivated planet in 2010: 2. the global gridded agricultural production maps. Earth System Science Data 12, 3545–3572 (2020).
29. Sacks, W. J., Deryng, D., Foley, J. A. & Ramankutty, N. Crop planting dates: an analysis of global patterns. Global ecology and biogeography, 19(5), 607-620 (2010).
30. Mueller, N. D., Gerber, J. S., Johnston, M., Ray, D. K., Ramankutty, N. & Foley, J. A. Closing yield gaps through nutrient and water management. Nature 490(7419), 254-257 (2012).
31. Brandt, M., Rasmussen, K., Hiernaux, P., Herrmann, S., Tucker, C. J., Tong, X., Tian, F., Mertz, O., Kergoat, L., Mbow, C. & David, J. L. Reduction of tree cover in West African woodlands and promotion in semi-arid farmlands. Nature Geoscience 11(5), 328-333 (2018).
32. Cotillon, S.E. & Tappan, G.G. Landscapes of West Africa—A Window on a Changing World; U.S. Geological Survey EROS: Garretson, SD, USA (2016).
33. Gilbert, M., Nicolas, G., Cinardi, G., Van Boeckel, T. P., Vanwambeke, S. O., Wint, G. W. & Robinson, T. P. Global distribution data for cattle, buffaloes, horses, sheep, goats, pigs, chickens and ducks in 2010. Scientific Data 5(1), 1-11 (2018).
34. Robinson, T. P., Thornton, P. K., Franceschini, G., Kruska, R. L., Chiozza, F., Notenbaert, A. M. O., Cecchi, G., Herrero, M. T., Epprecht, M., Fritz, S. & You, L. Global livestock production systems. FAO and ILRI (2011).
35. ACF. Rapport de Biomasse Sahel, Le programme de surveillance pastorale est basé au Bureau Régional de l’Afrique Centrale et de l’Ouest d’Action Contre la Faim, Access date: 2020-11-20. URL: https://sigsahel.info/ (2020).
36. Gibbs, H. K. & Salmon, J. M. Mapping the world's degraded lands. Applied geography 57, 12-21 (2015).
